# PKA inhibits WNT signalling in adrenal cortex zonation and prevents malignant tumour development

**DOI:** 10.1038/ncomms12751

**Published:** 2016-09-14

**Authors:** Coralie Drelon, Annabel Berthon, Isabelle Sahut-Barnola, Mickaël Mathieu, Typhanie Dumontet, Stéphanie Rodriguez, Marie Batisse-Lignier, Houda Tabbal, Igor Tauveron, Anne-Marie Lefrançois-Martinez, Jean-Christophe Pointud, Celso E. Gomez-Sanchez, Seppo Vainio, Jingdong Shan, Sonia Sacco, Andreas Schedl, Constantine A. Stratakis, Antoine Martinez, Pierre Val

**Affiliations:** 1 CNRS, UMR 6293, GReD, Inserm U1103, Clermont Université, F-63171 Aubière Cedex, France; 2 Developmental Endocrine Oncology and Genetics, Section on Genetics and Endocrinology, Eunice Kennedy Shriver National Institute of Child Health and Human Development, Bethesda, Maryland 20892-1103, USA; 3 Centre Hospitalier Universitaire, Service d'Endocrinologie, Faculté de Médecine, F-63000 Clermont-Ferrand, France; 4 Division of Endocrinology, G.V. (Sonny) Montgomery VA Medical Center, Jackson, Mississippi 39216, USA; 5 Department of Medicine-Endocrinology, University of Mississippi Medical Center, Jackson, Mississippi 39216, USA; 6 Biocenter Oulu, Laboratory of Developmental Biology, InfoTech Oulu, Center for cell Matrix Research, Faculty of Biochemistry and Molecular Medicine, University of Oulu, 90220 Oulu, Finland; 7 Inserm UMR1091, CNRS UMR 7277, Institute of Biology Valrose, F-06108 Nice, France

## Abstract

Adrenal cortex physiology relies on functional zonation, essential for production of aldosterone by outer zona glomerulosa (ZG) and glucocorticoids by inner zona fasciculata (ZF). The cortex undergoes constant cell renewal, involving recruitment of subcapsular progenitors to ZG fate and subsequent lineage conversion to ZF identity. Here we show that WNT4 is an important driver of WNT pathway activation and subsequent ZG differentiation and demonstrate that PKA activation prevents ZG differentiation through WNT4 repression and WNT pathway inhibition. This suggests that PKA activation in ZF is a key driver of WNT inhibition and lineage conversion. Furthermore, we provide evidence that constitutive PKA activation inhibits, whereas partial inactivation of PKA catalytic activity stimulates β-catenin-induced tumorigenesis. Together, both lower PKA activity and higher WNT pathway activity lead to poorer prognosis in adrenocortical carcinoma (ACC) patients. These observations suggest that PKA acts as a tumour suppressor in the adrenal cortex, through repression of WNT signalling.

## Abstract (web summary)

The adrenal cortex undergoes functional zonation to generate an outer zona glomerulosa (ZG) and inner zona fasciculata (ZF), but how this is regulated at a molecular level is unclear. Here, the authors show that ZG differentiation is stimulated by WNT signalling and that PKA blocks WNT signalling to allow ZF differentiation and also prevents WNT-induced cancer development.

## Graphical abstract

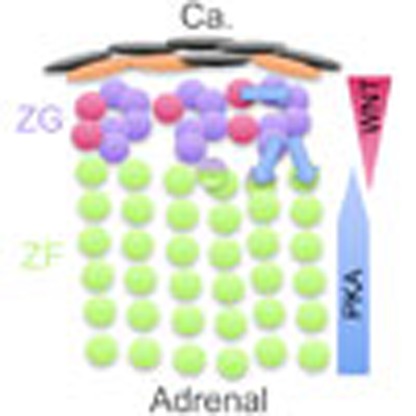

The adrenal cortex plays essential roles in body homeostasis through secretion of mineralocorticoids, essential for sodium and potassium homeostasis and glucocorticoids, which are involved in stress response, glucose homeostasis and immune suppression. The production of these two distinct classes of steroids is the result of functional adrenal cortex zonation in the perinatal period^1^. This corresponds to the formation of two concentric layers of differentiated cells within mouse adrenal cortex. The outermost zona glomerulosa (ZG) produces mineralocorticoids in response to Angiotensin II stimulation whereas the innermost zona fasciculata (ZF) synthesizes glucocorticoids in response to pituitary ACTH. Although the establishment and maintenance of functional zonation is essential for proper adrenal function, the molecular mechanisms involved in these processes remain unclear^2^. Postnatal adrenal cortex undergoes constant cell renewal. Recent lineage tracing studies have shown that adrenal cortex cell progenitors are recruited from capsular/subcapsular reservoirs through Shh/Gli1 signalling^3^,^4^. These cells migrate centripetally and contribute to both ZG and ZF renewal after differentiation. Interestingly, lineage-tracing experiments with the regulatory regions of *Cyp11b2* have shown that the preferred pathway for adrenal renewal involves initial differentiation to ZG and subsequent lineage conversion to ZF, along centripetal cell migration^4^. This suggests that cells receive positional cues leading them to sequentially differentiate as ZG and ZF cells along their migration. In the liver, spatial restriction of WNT signalling pathway activation plays an essential role in functional zonation^5^. In the adrenal cortex, WNT signalling activity is essentially restricted to ZG and we showed that loss of this restriction in genetic models results in ectopic ZG differentiation within ZF. *In vivo* and *in vitro*, β-catenin activation is also associated with upregulation of AT1R and CYP11B2, two key determinants of ZG identity^6^,^7^. These data strongly suggest that WNT/β-catenin is a master driver of ZG identity that has to be inhibited to allow lineage conversion from ZG to ZF.

Expression of MC2R, the receptor for ACTH and MRAP, an accessory protein essential to trigger cAMP/PKA signalling pathway activation is higher in ZF than in ZG^8^ and MC2R has been shown to play an essential role in ZF differentiation^9^. Consistent with its role in the establishment of ZG identity β-catenin activation can repress expression of MC2R and of one of its ZF-specific targets, CYP11B1 (ref. 10).

In this paper, we postulated that reciprocally, cAMP/PKA signalling played a role in repressing WNT/β-catenin signalling to allow lineage conversion and functional adrenal cortex zonation. Indeed, by a combination of hormonal manipulations and genetic alterations, we show that PKA activation prevents ZG differentiation through WNT4 repression and WNT pathway inhibition. This suggests that PKA activation in ZF is a key driver of WNT inhibition and lineage conversion. We further demonstrate that PKA activation is able to restrain WNT-mediated malignant adrenal cortex tumorigenesis. These data suggest an all-encompassing role of PKA-dependent WNT signalling inhibition in adrenal homeostasis and disease.

## Results

### PKA activation inhibits WNT signalling and ZG differentiation

**Figure 1:**
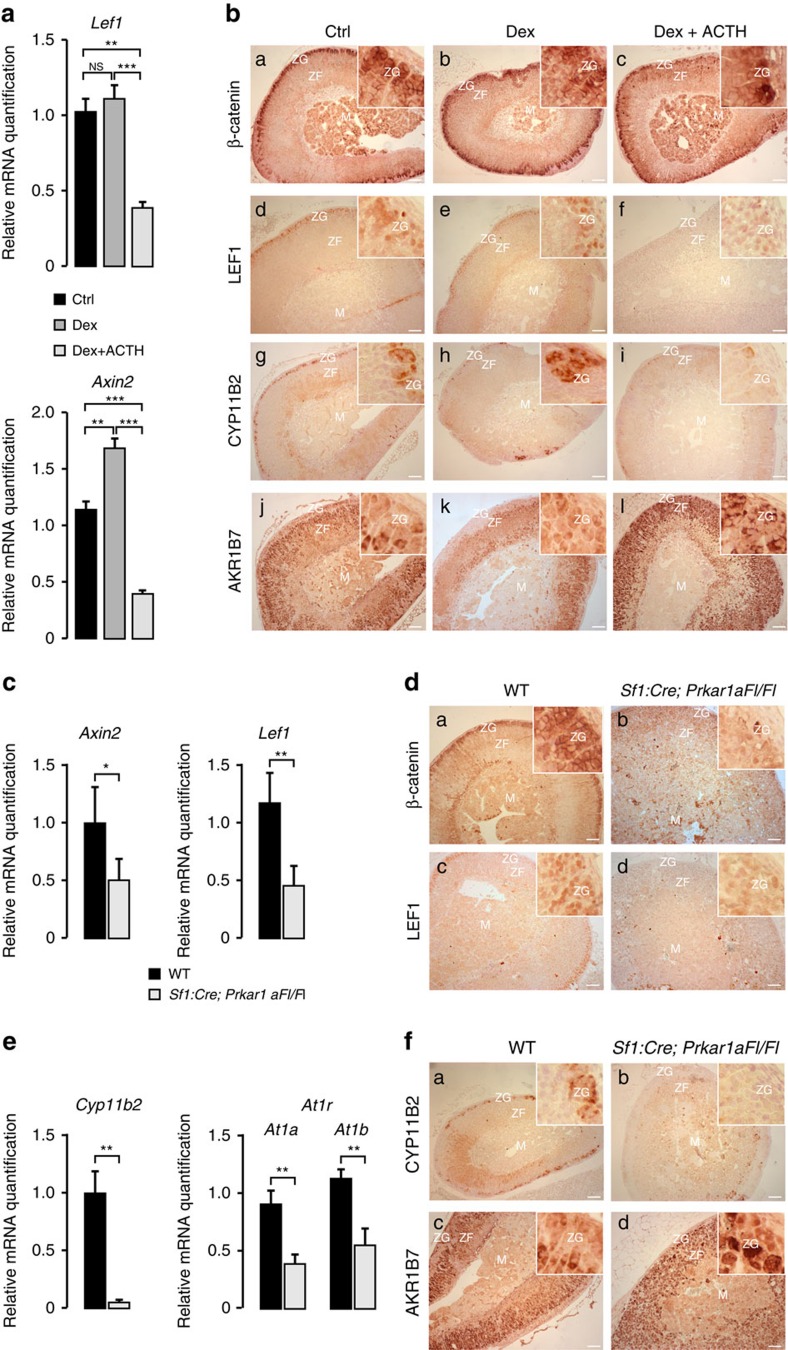
Antagonistic effects of PKA on WNT signalling and ZG differentiation. (**a**) ACTH treatment inhibits WNT target genes expression in the adrenal gland. Wild-type C57/Bl6 mice were treated for 5 days with vehicle (Ctrl) or Dexamethasone (Dex) or for 3 days with dexamethasone followed by 2 days of Synacthene retard (Dex+ACTH). *Lef1* and *Axin2* expression levels were then analysed by RT–qPCR on mRNAs extracted from the adrenals. Bars represent mean expression in four animals per group±s.e.m. (**b**) WNT pathway and ZG differentiation are inhibited by ACTH treatment. Expression of β-catenin, LEF1 (WNT pathway), AKR1B7 (ZF) and CYP11B2 (ZG) was analysed by immunohistochemistry in the adrenals from the same animal groups as in **a**. (**c**) Constitutive activation of PKA signalling inhibits WNT target genes expression in the adrenal gland. *Axin2* and *Lef1* expression levels were analysed by RT–qPCR on mRNAs extracted from wild-type and *Sf1:Cre;Prkar1aFl/Fl* adrenals. (**d**) Constitutive activation of PKA signalling inhibits β-catenin and LEF1 accumulation in ZG. Expression of β-catenin and LEF1 was analysed by immunohistochemistry in the adrenals from wild-type and *Sf1:Cre;Prkar1aFl/Fl* adrenals. (**e**) ZG differentiation is inhibited by constitutive PKA signalling. *Cyp11b2* and angiotensin II receptor *AT1R* isoforms a and b expression levels were analysed by RT–qPCR on mRNAs extracted from wild-type and *Sf1:Cre;Prkar1aFl/Fl* adrenals. (**f**) Constitutive PKA signalling promotes ZF differentiation at the expense of ZG. Expression of CYP11B2 and AKR1B7 was analysed by immunohistochemistry in the adrenals from wild-type and *Sf1:Cre;Prkar1aFl/Fl* adrenals. In **c**,**e**, bars represent mean expression in six animals per group±s.e.m. Statistical analyses were conducted by one-way ANOVA followed by Tukey's *post hoc* test (**a**) or by Student's *t*-test (**c**,**e**). *P<0.05; **P<0.005; ***P<0.0005; NS, not significant. In all the images, insets are focused on ZG. Scale bars, 100 μm. M, medulla; ZG, zona glomerulosa; ZF, zona fasciculata.

To evaluate a potential role of ACTH/cAMP signalling in repressing WNT/β-catenin pathway, we injected adult wild-type female mice with dexamethasone, a synthetic glucocorticoid alone for 5 days (subcutaneously twice daily, 75 μg per mouse) to block endogenous ACTH secretion or dexamethasone for 3 days, followed by ACTH for 2 days to evaluate the effect of exogenous ACTH administration (intramuscularly, Synacthene retard 1.2 U). As expected, the expression of *Akr1b7,* an ACTH/cAMP/PKA responsive ZF differentiation marker^11^, was significantly reduced by dexamethasone and markedly induced by ACTH treatment (analysis of variance (ANOVA), Supplementary Fig. 1A). In contrast, reverse transcription quantitative PCR (RT–qPCR) analyses showed a significant decrease in the expression of both *Lef1* and *Axin2*, two canonical WNT target genes, in ACTH-treated animals (ANOVA, Fig. 1a). Conversely, *Axin2* expression was significantly increased in the animals treated with dexamethasone (ANOVA, Fig. 1a). *In situ* analysis of β-catenin expression showed no major differences between the three treatment groups (Fig. 1b, a–c). However, ACTH treatment resulted in a robust reduction of LEF1 expression, which was almost completely extinguished in the ZG of treated animals (Fig. 1b, f versus d). This was further confirmed by extinction of LacZ activity in the adrenals of TopGal WNT signalling reporter mice^12^ that were treated for 3 days with ACTH (Supplementary Fig. 1B). Altogether these data suggested that ACTH treatment could result in inhibition of WNT signalling in the adrenal cortex. We then sought to determine the impact of ACTH on ZG differentiation by immuno-histochemistry. This showed decreased CYP11B2 expression (Fig. 1b, i versus g) and a concomitant expansion of the expression domain of the ZF marker AKR1B7 within ZG (Fig. 1b, l versus j and Supplementary Fig. 1C). Altogether, these observations showed that stimulation of ACTH signalling pathway antagonized WNT signalling within the adrenal cortex, which was correlated with inhibition of ZG and expansion of ZF differentiation in WT adrenals. However, these experiments relied on short-term treatments and were performed on tissues with already established zonation. ACTH signalling in the adrenal relies on cAMP production and stimulation of PKA. Therefore, to further establish a potential role of ACTH/PKA-mediated WNT inhibition in the establishment of adrenal cortex zonation, we devised a genetic model of developmental constitutive PKA activation. For this, mice bearing a floxed allele of *Prkar1a*^13^ were mated with mice expressing Cre recombinase under the control of Sf1 regulatory regions, which allows recombination as early as E10.5 (ref. 14). Analysis of recombination with the mTmG reporter system^15^ confirmed that Sf1 regulatory regions drove robust recombination throughout the adrenal cortex, including ZG as shown by co-expression of Cyp11b2 and GFP (Supplementary Fig. 1D). RT–qPCR analysis of *Prkar1a* mRNA accumulation showed robust downregulation in the adrenals of *Sf1:Cre;Prkar1aFl/Fl* mice compared with controls, which confirmed efficient inactivation of *Prkar1a* (Supplementary Fig. 1E). This was accompanied by upregulation of *Akr1b7* (Supplementary Fig. 1F) and *StAR* (Supplementary Fig. 1G) mRNA accumulation (two known targets of PKA) and centrifugal expansion of fetal-like 20αHSD-positive cells (Supplementary Fig. 1H,I). This was reminiscent of AdKO mice in which, *Prkar1a* inactivation was driven by *Akr1b7* regulatory regions^16^ (These mice will be fully characterized in another report). We then evaluated WNT signalling by RT–qPCR. Consistent with the effect of ACTH stimulation, chronic activation of PKA signalling resulted in robust repression of *Axin2* and *Lef1* mRNA accumulation (Fig. 1c). This was confirmed by a marked decrease in β-catenin and LEF1 protein expression in the outer cortex of *Sf1:Cre;Prkar1aFl/Fl* mice compared with wild-type mice (Fig. 1d, a–d). Consistent with the hypothesis that PKA-mediated WNT inhibition resulted in deficient ZG differentiation, RT–qPCR showed a marked downregulation of *Cyp11b2* and Angiotensin II receptors *At1a/At1b* expression in *Sf1:Cre;Prkar1aFl/Fl* animals (Fig. 1e). This was confirmed *in situ* by an almost complete extinction of CYP11B2 expression (Fig. 1f, a,b) and expansion of AKR1B7 towards the presumptive ZG (Fig. 1f, c,d and Supplementary Fig. 1J), which was consistent with increased *Akr1b7* mRNA accumulation (Supplementary Fig. 1F). In line with the *in situ* phenotype, intra-adrenal aldosterone levels were significantly decreased in *Sf1:Cre;Prkar1a Fl/Fl* animals (*t*-test, Supplementary Fig. 1K). We thus concluded that chronic constitutive activation of PKA throughout the adrenal cortex resulted in WNT signalling repression and expansion of ZF at the expense of ZG.

### PKA activation induces inactivating β-catenin phosphorylation

**Figure 2:**
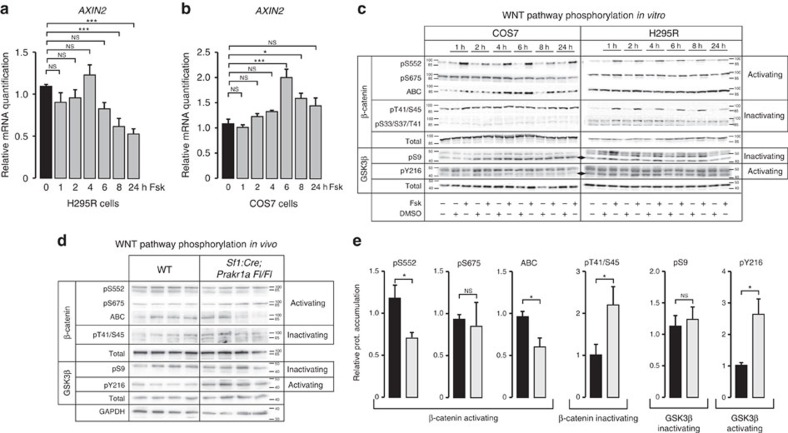
Effect of PKA activation on downstream WNT pathway phosphorylation. (**a**) Forskolin represses *AXIN2* expression in human adrenocortical H295R cells. Human H295R cells were treated with either vehicle (0, DMSO) or Forskolin 10^−5^ M (Fsk) for 1 to 24 h and *AXIN2* expression was analysed by RT–qPCR. Bars represent mean expression in at least three independent experiments (in triplicate)±s.e.m. Statistical analyses were performed by one-way ANOVA followed by Dunnett's multiple comparison test. (**b**) Forskolin activates *AXIN2* expression in COS7 cells. COS7 cells were treated with Forskolin for 1 to 24 h and *AXIN2* expression was analysed as described in **a**. (**c**) Differential phosphorylation of β-catenin and GSK3β in COS7 and H295R cells. COS7 and H295R cells were treated as in **a**. Proteins were extracted and subjected to western blot analysis with antibodies to phospho/dephospho-β-catenin, phospho-GSK3β or the corresponding total proteins. β-catenin phosphorylations/dephosphorylations were sorted as activating (pS552, pS675, active beta-catenin) and inactivating (pT41/S45 and pS33/S37/T41). GSK3β serine 9 phosphorylation (pS9) results in its inactivation. GSK3β tyrosine 216 phosphorylation (pY216) results in its activation. Phospho-GSK3 antibodies recognize phospho-GSK3β arrowheads, and phospho-GSK3α (top band) (**d**) Phosphorylation of β-catenin and GSK3β in response to constitutive PKA activation *in vivo*. Activating and inactivating phosphorylations of β-catenin and Gsk3β were analysed in adrenal protein extracts from four wild-type and 4 *Sf1:Cre;Prkar1Fl/Fl* mice as described in **c**. (**e**) Constitutive activation of PKA inhibits activating phosphorylations and stimulates inactivating phosphorylations *in vivo.* Graphs show pooled quantification of phospho-specific signals in **d** and Supplementary Fig. 3, representing a total of nine wild-type and nine *Sf1:Cre;Prkar1aFl/Fl* mice. These were normalized to quantification of total corresponding proteins. Statistical analyses were performed with Student's *t*-test. *P<0.05; ***P<0.0005; NS, not significant.

In contrast with our data, previously published studies suggested that PKA signalling pathway activation was associated with WNT pathway activation in a number of cell types and tissues^17^,^18^,^19^,^20^. We thus wanted to identify the molecular mechanisms involved in this unexpected inhibitory interaction in the adrenal cortex. As a first approach, we used H295R human adrenocortical cell lines and COS7 cells, in which WNT pathway responds positively to PKA stimulation^18^. As expected, the treatment of H295R cells with Forskolin, a potent stimulator of adenylate cyclase, resulted in increased *StAR* expression (Supplementary Fig. 2A). Consistent with our *in vivo* data, Forskolin treatment repressed *Axin2* expression in H295R cells (Fig. 2a). In contrast, a similar treatment resulted in *Axin2* induction in COS7 cells (Fig. 2b). Data from the literature suggest that PKA can activate β-catenin by direct phosphorylation on serine residues 552 and 675, which induces its nuclear localization and transcriptional activity. PKA can also indirectly stimulate β-catenin accumulation by phosphorylating GSK3β serine 9, which results in its inactivation^17^,^18^,^19^,^20^. As expected, pS552 was rapidly increased in response to Forskolin treatment in COS7 cells (Fig. 2c, Supplementary Fig. 2B). This was associated with a transient increase in accumulation of active dephospho-β-catenin (ABC, Fig. 2c, Supplementary Fig. 2D). However, we did not observe significant induction of activating β-catenin serine 675 phosphorylation or of inactivating GSK3β serine 9 phosphorylation (ANOVA, Fig. 2c, Supplementary Fig. 2C,G). Consistent with the activated status of the pathway, phosphorylation of the residues that target β-catenin for proteasomal degradation (S33/S37/S45/T41) was not increased in response to Forskolin (Fig. 2c, Supplementary Fig. 2E,F). In H295R cells, Forskolin also induced phosphorylation of S552 as efficiently as in COS7 cells (Fig. 2c, Supplementary Fig. 2I). However, this was also associated with a robust increase in inactivating pT41/S45 (Fig. 2c, Supplementary Fig. 2L) even though pS33/S37/T41 phosphorylation was not altered (Fig. 2c, Supplementary Fig. 2M). Consistent with this, there was also no accumulation of the active ABC β-catenin in response to Forskolin in H295R cells (Fig. 2c, Supplementary Fig. 2K). Analysis of activating GSK3β tyrosine 216 phosphorylation, showed no effect of Forskolin in both cell lines (Fig. 2c, Supplementary Fig. 2H,O). Although pS33/S37/T41 β-catenin was not detectable *in vivo* (not shown), these *in vitro* findings were further confirmed by a significant increase in pT41/S45 β-catenin accumulation in the adrenals of *Sf1:Cre;Prkar1aFl/Fl* mice compared with their wild-type littermates (*t*-test, Fig. 2d,e, Supplementary Fig. 3), which was associated with a concomitant decrease in pS552 activating phosphorylation (Fig. 2d,e, Supplementary Fig. 3) and of active ABC β-catenin accumulation (Fig. 2d,e, Supplementary Fig. 3). In contrast, inactivating GSK3β S9 phosphorylation was not altered in response to *Prkar1a* inactivation (Fig. 2d,e, Supplementary Fig. 3). However, there was a significant increase in activating GSK3β tyrosine 216 phosphorylation^21^, which could account for increased inactivating β-catenin T41/S45 phosphorylation (*t*-test, Fig. 2d,e, Supplementary Fig. 3). We thus concluded that in contrast with observations in COS7 cells, PKA stimulation was associated with an increase in inactivating and a decrease in activating β-catenin phosphorylation in adrenocortical cells *in vivo*.

### Deregulation of WNT pathway regulators upon PKA activation

**Figure 3:**
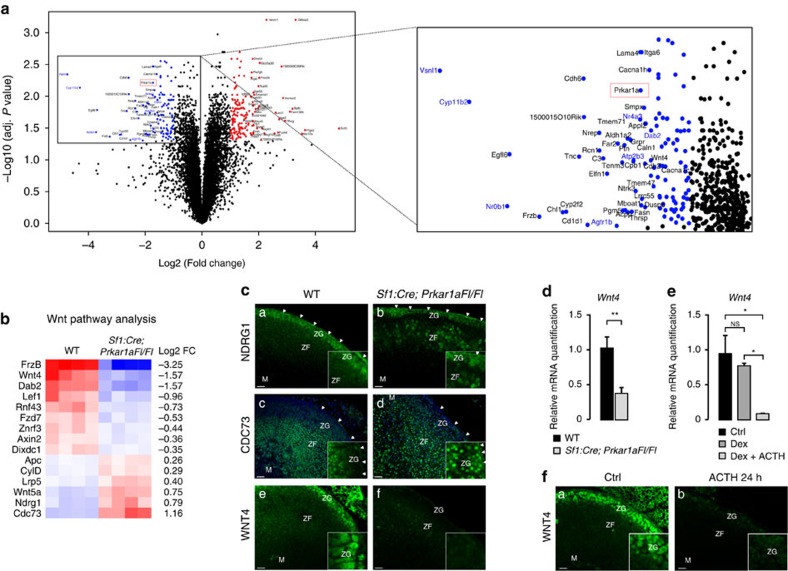
WNT4 is downregulated by PKA signalling activation. (**a**) Constitutive activation of PKA signalling inhibits ZG markers expression. Volcano plot shows differential gene expression in adrenals from four *Sf1:Cre;Prakr1aFl/Fl* mice compared with four wild-type littermates. A number of ZG differentiation markers (gene symbols in blue) are among the most significantly downregulated genes. As expected, *Prkar1a* expression (red rectangle) is significantly decreased in knockouts. (**b**) Constitutive activation of PKA signalling modulates expression of WNT pathway target genes and regulators. Colour-coded heatmap shows expression of the 15 genes that were significantly deregulated (adjusted *P* value <0.05). (**c**) Expression of NDRG1, CDC73 and WNT4 in mouse adrenals. Expression of the two potential WNT signalling repressors NDRG1 and CDC73 and of the WNT ligand WNT4 was analysed by immunohistochemistry in wild-type (*n*=4; a,c,e) and *Sf1:Cre;Prkar1aFl/Fl* adrenals (*n*=4; b,d,f). Hoechst nuclear staining in c and d highlights the lack of Cdc73 expression in wild-type outer cortex. Insets show high magnification pictures of ZG. Arrowheads show adrenal capsule. (**d**) Constitutive activation of PKA signalling inhibits *Wnt4* expression. *Wnt4* expression levels were analysed by RT–qPCR on mRNAs extracted from wild-type and *Sf1:Cre;Prkar1aFl/Fl* adrenals. Bars represent the mean expression in six animals per group±s.e.m. Statistical analyses were performed by Student's *t*-test. (**e**) ACTH stimulation represses *Wnt4* expression. *Wnt4* expression levels were analysed by RT–qPCR on mRNAs extracted from adrenals of wild-type mice treated with vehicle, dexamethasone or dexamethasone and ACTH as in Fig. 1a. Bars represent the mean expression in four animals per group±s.e.m. Statistical analyses were performed by one-way ANOVA followed by Tukey's *post hoc* test. (**f**) ACTH induces a rapid decrease in WNT4 protein accumulation. WNT4 expression was analysed by immunohistochemistry in the adrenal cortex of wild-type mice (*n*=4), 24 h after a single ACTH (Synacthene retard) injection. *P<0.05; **P<0.005. Scale bars, 100 μm.

**Figure 4:**
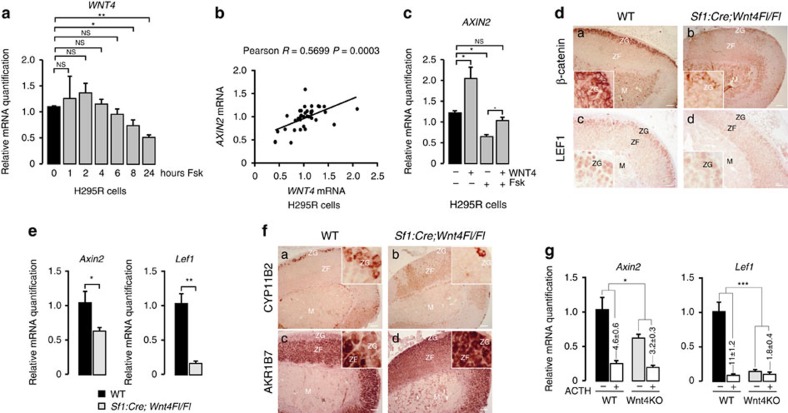
WNT4 repression mediates the effect of PKA on WNT signalling in the adrenal gland. (**a**) Forskolin represses *WNT4* expression in human adrenocortical H295R cells. Human H295R cells were treated with either vehicle (0) or Forskolin 10^−5^M (Fsk) for 1 to 24 h and *WNT4* expression was analysed by RTqPCR. Bars represent mean expression in three independent experiments (in triplicate)±s.e.m. (**b**) *AXIN2* and *WNT4* expression levels are correlated in H295R cells. *AXIN2* and *WNT4* expression levels were correlated on the basis of RTqPCR data generated from H295R cell treatments. (**c**) Overexpression of WNT4 reverses the effect of Forskolin on *AXIN2* expression. H295R cells were transfected with pCMV-WNT4 to induce overexpression of WNT4 and treated with DMSO or Forskolin 10^−5^M (Fsk) for 8 h. Expression of *AXIN2* was determined by RTqPCR. Bars represent mean expression in three independent experiments±s.e.m. Statistical analyses were performed by one-way ANOVA followed by Tukey's post-hoc test (**d**) *Wnt4* genetic ablation inhibits WNT pathway activation in ZG. Expression of β-catenin and WNT pathway target LEF1 was analysed by immunohistochemistry in the adrenals from wild-type and *Sf1:Cre;Wnt4Fl/Fl mice*. (**e**) *Wnt4* genetic ablation is associated with decreased *Axin2* and *Lef1* expression in the adrenal. *Axin2* and *Lef1* expression levels were analysed by RTqPCR on mRNAs extracted from wild-type and *Sf1:Cre;Wnt4Fl/Fl* adrenals. Bars represent the mean expression in 5 animals per group±s.e.m. Statistical analyses were performed by Student's *t* test. (**f**) *Wnt4* genetic ablation is associated with loss of ZG differentiation and expansion of ZF. Expression of CYP11B2 (ZG) and AKR1B7 (ZF) was analysed in the adrenals from wild-type and *Sf1:Cre;Wnt4Fl/Fl* mice. (**g**) The ability of ACTH to repress *Axin2* and *Lef1* expression is blunted in Wnt4 knockout adrenals. Wild-type and *Sf1:Cre;Wnt4Fl/Fl* mice were treated for three days with PBS or ACTH (Synacthene Retard) and *Axin2* and *Lef1* expression levels were evaluated by RTqPCR. Bars represent the mean expression in 5 adrenals per genotype and per treatment group±s.e.m. Fold repression by ACTH over PBS in both wild-type and *Sf1:Cre;Wnt4Fl/Fl* mice is shown. Statistical analyses were performed by Student's *t* test. *P<0.05; **P<0.005; ***P<0.0005; NS: Not significant. Scale bars, 100 μm. ZG, zona glomerulosa; ZF, zona fasciculata; M, medulla.

To further gain insight into the underpinnings of these phenomena, we conducted a comparative microarray analysis of gene expression in four wild-type and four *Sf1:Cre;Prkar1aFl/Fl* adrenals. Five hundred and five genes were found downregulated and 713 genes were found upregulated in response to *Prkar1a* gene inactivation (Fig. 3a and Supplementary Data 2). Consistent with inhibition of ZG differentiation in this model, a number of ZG markers such as *Cyp11b2, Agtr1b*, *Nr0b1*, *Nr4a3*, *Atp2b3*, *Vsnl1* and *Dab2* were among the most significantly downregulated genes in knockout adrenals compared with wild-type (adjusted *t*-test, Fig. 3a, right panel). To gain insight into WNT pathway regulation, we extracted expression levels of a list of 127 WNT targets and regulators (Supplementary Data 1). Fifteen of these 127 genes were significantly deregulated (adjusted *t*-test, adjusted *P* value <0.05) in knockout compared with wild-type adrenals (Fig. 3b), which was confirmed by RT–qPCR conducted on six independent WT and six knockout samples (Supplementary Fig. 4A,B). Consistent with our findings (Fig. 1), this unbiased analysis showed downregulation of WNT target genes *Lef1* and *Axin2*, but also of *Dab2* (ref. 22), *Rnf43* and *Znrf3* (ref. 23). We then evaluated which of these genes could account for decreased WNT signalling in response to PKA stimulation. The two most upregulated genes—*Ndrg1* and *Cdc73*—encode potential repressors of WNT signalling. NDRG1 is a metastasis suppressor that induces localization of dephosphorylated β-catenin at the plasma membrane and inhibits β-catenin nuclear translocation by reducing nuclear localization of PAK4 (ref. 24). *Cdc73* encodes parafibromin, a tumour suppressor that has been shown to work either as a WNT pathway activator^25^ or repressor depending on its phosphorylation status^26^. Immunohistochemical analysis showed expression of NDRG1 in the adrenal capsule and ZG in wild-type mice (Fig. 3c,a and Supplementary Fig. 5A). Consistent with micro-array data, there was increased expression of NDRG1 in *Sf1:Cre;Prkar1aFl/Fl* adrenals (Fig. 3c,b). However, the increase was mostly restricted to ZF, suggesting that it was unlikely to account for WNT pathway repression within ZG. Interestingly, although CDC73 was mostly accumulated in the nucleus of ZF cells in wild-type adrenals (Fig. 3c,c and Supplementary Fig. 5A), its expression domain expanded within the presumptive ZG in *Sf1:Cre; Prkar1a Fl/Fl* adrenals (Fig. 3c,d). This suggested that CDC73 could participate in PKA-mediated WNT pathway repression. Consistent with this idea, there was a significant negative correlation between *Cdc73* and *Lef1* or *Axin2* expression levels in RT–qPCR experiments (Pearson's correlation, Supplementary Fig. 4C). *FrzB*, the most downregulated gene in our analysis (Fig. 3b), encodes the WNT signalling repressor SFRP3. It is thus unlikely to be responsible for WNT pathway repression in response to PKA activation. However, there was also marked downregulation of the WNT ligand *Wnt4* (Fig. 3b). Mice with constitutive *Wnt4* inactivation have decreased *Cyp11b2* expression and plasma aldosterone concentrations at birth^27^. Conversely, WNT4 overexpression in human adrenocortical cell lines induces CYP11B2 expression and aldosterone production^28^. This suggested that WNT4 could be one of the drivers of WNT pathway activation in the adrenal cortex and of subsequent ZG differentiation. Immunohistochemical analysis showed WNT4 expression within ZG in wild-type adrenals, which was almost completely extinguished in *Sf1:Cre;Prkar1aFl/Fl* mice (Fig. 3c, f versus e and Supplementary Fig. 5A). Consistent with our microarray data, RT–qPCR analysis of *Wnt4* expression showed a marked inhibition in mice with genetic ablation of *Prkar1a* (Fig. 3d and Supplementary Fig. 4A,B) and in wild-type mice in response to ACTH treatment following dexamethasone inhibition of endogenous ACTH secretion (Fig. 3e). This suggested that decreased *Wnt4* expression could account for decreased WNT signalling in response to PKA stimulation. In support for this hypothesis, *Wnt4* and *Axin2* expression levels were significantly correlated in both models (Pearson's correlation, Supplementary Fig. 4C and Supplementary Fig. 5B,C). To rule out a possible reduction in the number of WNT4 expressing cells in animal models in response to long-term PKA stimulation, we performed a single injection of ACTH (Synacthene retard) and analysed WNT4 expression 24 h later. This also showed a robust decrease in WNT4 protein (Fig. 3f, b versus a) and mRNA accumulation (Supplementary Fig. 5D). Consistent with a rapid effect of PKA signalling on WNT4 expression, *WNT4* mRNA accumulation was also significantly reduced after 8 h Forskolin treatment in H295R cells (ANOVA, Fig. 4a), which was correlated with *AXIN2* expression (Fig. 4b). Conversely, treatment with H89, a pharmacological PKA inhibitor, induced a modest increase in basal *WNT4* expression and prevented inhibition of *WNT4* expression by Forskolin treatment (Supplementary Fig. 5E). The potential role of *WNT4* repression in mediating the inhibitory effect of PKA on WNT signalling was further suggested by the observation that transfection of a plasmid encoding WNT4, counteracted the inhibitory effect of Forskolin stimulation on *AXIN2* expression in H295R cells (Fig. 4c).

### WNT4 repression is involved in WNT pathway inhibition by PKA

Altogether, these experiments suggested that WNT4 played an important role in WNT pathway stimulation in the adrenal cortex and that PKA-mediated WNT signalling repression may partly rely on WNT4 repression. To further confirm these hypotheses, we generated a mouse model of *Wnt4* inactivation within the adrenal cortex by mating *Wnt4Fl/Fl* mice^29^ with *Sf1:Cre* mice. As expected, this resulted in extinction of WNT4 expression in knockout adrenals (Supplementary Fig. 5A,F). Interestingly, mice with *Wnt4* inactivation had markedly reduced β-catenin (Fig. 4d, b versus a) and LEF1 (Fig. 4d, d versus c) accumulation within ZG. This was correlated with a significant reduction in *Axin2* and *Lef1* mRNA accumulation (*t-*test, Fig. 4e), which demonstrated that WNT4 was involved in canonical WNT pathway activation in the adrenal cortex. Consistent with the essential role of β-catenin in ZG differentiation, there was a marked reduction in the number of CYP11B2 positive cells in response to *Wnt4* inactivation (Fig. 4f, b versus a). This was further associated with expansion of ZF differentiation as shown by strong AKR1B7 expression within the presumptive ZG of mutant adrenals (Fig. 4f, d versus c and Supplementary Fig. 5G). Thus, *Wnt4* inactivation phenocopied the effect of constitutive PKA activation, resulting from genetic ablation of *Prkar1a* (compare Fig. 4d,f with Fig. 1d,f). To further demonstrate the involvement of WNT4 repression in PKA-mediated WNT signalling inhibition, we treated control littermates and *Sf1:Cre;Wnt4Fl/Fl* mice with ACTH (Synacthene retard) for 3 days. As expected, ACTH treatment resulted in robust repression of *Axin2* and *Lef1* mRNA accumulation in control adrenals (Fig. 4g). Interestingly, PKA-mediated *Lef1* repression was almost completely abrogated in *Wnt4* knockout adrenals (Fig. 4g). There was also a significant albeit less dramatic reduction in *Axin2* repression (*t*-test, Fig. 4g). Altogether, these data showed that WNT4 was an essential driver of ZG differentiation through WNT pathway activation and that *Wnt4* repression was, at least in part, responsible for WNT pathway inhibition in response to PKA activation.

### PKA activation inhibits WNT-induced tumorigenesis

**Figure 5:**
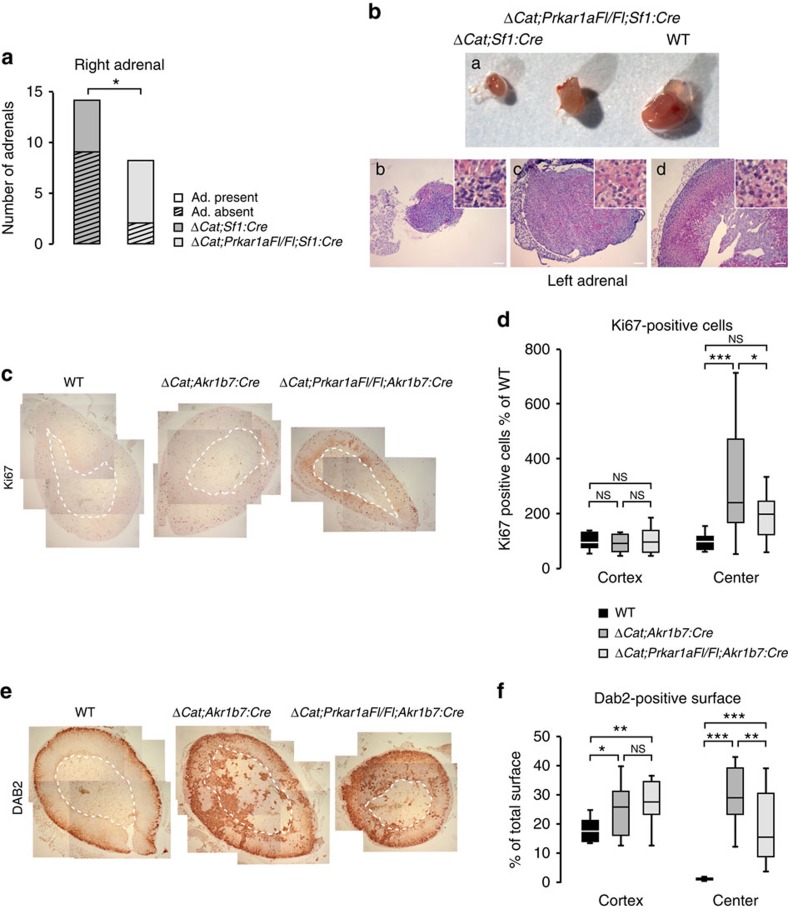
Constitutive PKA signalling counteracts constitutive WNT signalling. (**a**,**b**) Constitutive PKA activation partially rescues WNT-induced developmental phenotypes. (**a**) Constitutive activation of β-catenin with *Sf1:Cre* driver is associated with right adrenal gland aplasia in 9 out of 14 animals (left bar). Genetic ablation of *Prkar1a* significantly rescues this phenotype (aplasia in two out of eight animals, right bar), *P*=0.0416 in N-1 two proportion test. (**b**) Left adrenal size (a) and histological features are partially restored by genetic ablation of *Prkar1a* in the context of *Sf-1:Cre* mediated constitutive activation of β-catenin (c versus b). Wild-type adrenal is shown in d. (**c**,**d**) Constitutive PKA activation inhibits WNT-induced proliferation. (**c**) Ki67 expression was analysed by immunohistochemistry in wild-type, constitutive active β-catenin mutants (*ΔCat;Akr1b7:Cre*) and constitutive active β-catenin mutants in the context of *Prkar1a* genetic ablation (*ΔCat;Prkar1aFl/Fl;Akr1b7:Cre*). (**d**) Ki67 positive cells were separately counted in the adrenal cortex and central adrenal region of 12–14 animals of each genotype. Numbers of positive cells are represented as a percentage of positive cells in each wild-type compartment. (**e**,**f**) Constitutive PKA activation inhibits WNT-induced ectopic ZG differentiation. (**e**) Expression of the ZG marker DAB2 (see Supplementary Fig. 6B) was analysed by immunohistochemistry in the same genotypes as in **c**. (**f**) DAB2 stained areas were measured separately in the adrenal cortex and central adrenal region of 10–15 animals of each genotype. Stained areas are expressed as a percentage of total adrenal surfaces. Statistical analyses in **d**,**f** were conducted by one-way ANOVA followed by Tukey's *post hoc* test *P<0.05; **P<0.005; ***P<0.0005; NS, not significant. Scale bars, 100 μm. Dashed lines delimit cortex from central adrenal area (medulla and ectopic glomerulosa cells).

We have previously shown that constitutive WNT pathway activation initially resulted in ectopic ZG hyperplasia and eventually adrenal carcinoma development^6^,^7^. We thus wanted to evaluate the capacity of constitutive PKA signalling to inhibit WNT-induced ectopic ZG differentiation and aberrant proliferation. For this, we initially mated mice with *Sf1:Cre*-mediated *Prkar1a* inactivation with *Ctnnb1Fl(ex3)* mice^30^ (to generate *ΔCat;Sf1:Cre* mice). However, consistent with previously published data^31^, the robust activation of WNT signalling triggered by Sf1-driven Cre expression resulted in left adrenal hypoplasia and in most cases right adrenal agenesis in *ΔCat;Sf1:Cre* mice at one month of age, which precluded analysis of adrenal differentiation and proliferation (Fig. 5a,b, a). However, in this context, genetic ablation of *Prkar1a* allowed partial rescue of the WNT-induced developmental phenotype. This manifested as recovery of an hypoplastic right adrenal in most mice (Fig. 5a) and an increase in overall adrenal size (Figs 5b,a), even though adrenal cortex histology was still markedly disrupted in both *ΔCat;Sf1:Cre* and *ΔCat;Prkar1aFl/Fl*;*Sf1:Cre* mice (Fig. 5b, b–d). We thus resorted to using our previously characterized *Akr1b7:Cre* line, in which the later and stochastic expression of Cre recombinase bypasses β-catenin-dependent developmental phenotypes (Supplementary Fig. 6A). At 6 months, the adrenal phenotype in ΔCat mice was characterized by increased proliferation of steroidogenic cells within the central adrenal region (Figs 5c,d and ref. 6). This was associated with aberrant differentiation of DAB2-positive ZG cells (ref. 32 and Supplementary Fig. 6B) within ZF and central adrenal region (Fig. 5e,f). Interestingly, ablation of *Prkar1a* resulted in a decrease in both proliferation and aberrant differentiation of ZG cells within the central adrenal region (Fig. 5d,f). This suggested that constitutive PKA activation through *Prkar1a* gene ablation could inhibit WNT-induced hyperproliferation and ectopic ZG differentiation.

### PKA inactivation accelerates WNT-induced tumorigenesis

**Figure 6:**
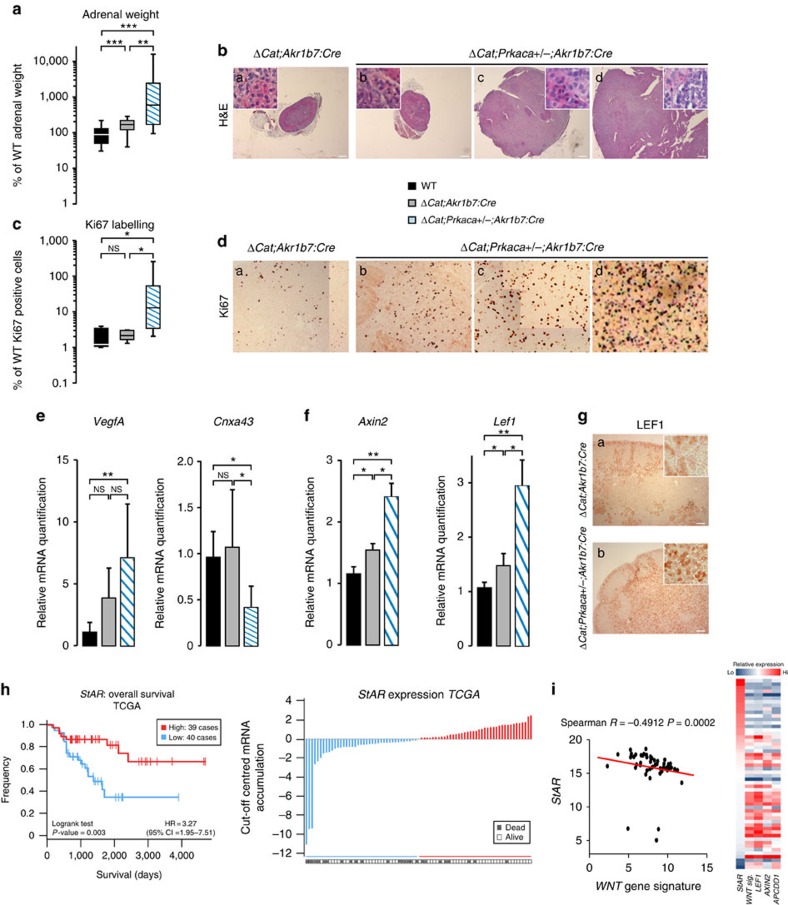
*Prkaca* heterozygosity accelerates WNT-induced tumorigenesis. (**a**) Adrenal weight is significantly increased by loss of one *Prkaca* allele. Adrenal glands from wild-type, *ΔCat;Akr1b7:Cre* and *ΔCat;Prkaca+/−;Akr1b7:Cre* mice were weighed on killing. (**b**) *Prkaca* heterozygosity accelerates WNT-induced hyperplasia and dysplasia. *ΔCat;Akr1b7:Cre* (a) and *ΔCat;Prkaca+/−;Akr1b7:Cre* (b–d) adrenals were counterstained with haematoxylin and eosin. Pictures in b–d represent the range of phenotypes resulting from *Prkaca* heterozygosity. Scale bar, 400 μm. (**c**,**d**) *Prkaca* heterozygosity increases proliferation. Ki67 positive cells were counted in five high-power fields per adrenal (WT, *n*=3; *ΔCat;Akr1b7:Cre, n*=4; *ΔCat;Prkaca+/−;Akr1b7:Cre*, *n*=9).Results are expressed as a labelling index. Pictures in **d** show high-power fields for one *ΔCat;Akr1b7:Cre* adrenal (a) and three different *ΔCat;Prkaca+/−;Akr1b7:Cre* adrenals (b–d). (**e**) *Prkaca* heterozygosity is associated with increased malignancy. *VegfA* and *Cnxa43* expression levels were analysed by RT–qPCR in wild-type (*n*=5), *ΔCat;Akr1b7:Cre* (*n*=7) and *ΔCat;Prkaca+/−;Akr1b7:Cre* (*n*=12) adrenals. (**f**,**g**) *Prkaca* heterozygosity increases WNT pathway activity. (**f**) *Axin2* and *Lef1* expression levels were analysed by RT–qPCR (**g**) LEF1 expression was analysed by immunohistochemistry in *ΔCat;Akr1b7:Cre* and *ΔCat;Prkaca+/−;Akr1b7:Cre* adrenals. Insets show ectopic accumulation of LEF1 within ZF. Scale bar, 100 μm. (**h**) Low *StAR* expression is associated with lower overall survival in ACC patients. The patients were separated as two equivalent groups of high (*n*=39) and low (*n*=40*) StAR* expression. Left panel: Kaplan–Meier estimates of overall survival. Right panel: patients were ordered by cut-off-centred *StAR* expression. Dark grey boxes indicate specific death. (**i**) WNT pathway activity is inversely correlated with *StAR* expression in ACC patients. Adrenocortical carcinoma patients with no mutation in *CTNNB1*, *APC and ZNRF3* were selected and a WNT pathway activation signature was established based on expression levels of *LEF1*, *AXIN2* and *APCDD1*. Left panel: Spearman correlation between WNT signature and *StAR* expression. Right panel: hierarchical clustering of patients as a function of *StAR* expression and corresponding levels of WNT gene signature and individual WNT target genes expression. Bars in **e**,**f** represent mean expression±s.e.m. Statistical analyses in **a**,**c**,**e** and **f** were conducted by one-way ANOVA followed by Tukey's *post hoc* *P<0.05; **P<0.005; ***P<0.0005; NS, not significant.

To further confirm the repressive action of PKA on WNT signalling, we decided to reduce catalytic PKA activity to assess a potential overactivation of WNT signalling. *Prkaca+/−* adrenals were indistinguishable from wild-type (Supplementary Fig. 6C), suggesting normal control of WNT signalling in this genetic context. However, combination of heterozygous *Prkaca* ablation with constitutive β-catenin activation resulted in a marked aggravation of WNT-induced phenotypes. Indeed, adrenal weight was markedly increased in 12-month-old *ΔCat;Prkaca+/−;Akr1b7:Cre* mice compared with their *ΔCat;Akr1b7:Cre* littermates (Fig. 6a). This was correlated with a more dysplastic histology (Fig. 6b, b–d versus a) and a significant increase in Ki67 labelling index (ANOVA, Fig. 6c,d). Acceleration of tumour development in response to *Prkaca* heterozygous ablation was further confirmed by a significant increase in *VegfA* and a decrease in *Connexinα43* expression, two hallmarks of adrenal malignancy (ANOVA, Fig. 6e). The correlation between decreased PKA activity and increased WNT signalling, was shown by a significant increase in *Axin2* and *Lef1* mRNA accumulation in *ΔCat;Prkaca+/−;Akr1b7:Cre* mice, compared with *ΔCat;Akr1b7:Cre* mice (ANOVA, Fig. 6f). This was further confirmed by a marked increase in the number of LEF1-labelled cells in immunohistochemistry (Fig. 6g, b versus a). These data showed that reduced PKA activity could favour malignant WNT-induced tumorigenesis. To evaluate a potential involvement of this interaction in the context of human ACC development, we took *StAR* expression as a proxy to PKA pathway activation, within the TCGA cohort of ACC^33^ (RNA sequencing data). Interestingly, patients with high *StAR* expression levels (that is, high PKA activity) had significantly higher overall survival, when compared with patients with low *StAR* accumulation (that is, low PKA activity; LogRank test, Fig. 6h). To establish a link with WNT signalling, we determined a WNT pathway activation signature based on the geometric mean of *LEF1*, *AXIN2* and *APCDD1* expression levels. Consistent with previously published data, patients with elevated WNT signalling had significantly lower survival than patients with low WNT signalling (Supplementary Fig. 7). To further confirm the association between low PKA signalling and high WNT signalling, we selected patients with no mutations in *CTNNB1* (activating), *APC* or *ZNRF3* (inactivating) and evaluated a potential negative association between *StAR* expression and WNT signature. Interestingly, WNT activation signature was inversely correlated with *StAR* (Fig. 6i, left panel). This correlation was further confirmed by clustering analysis of patients on the basis of *StAR* expression (Fig. 6i, right panel). Indeed, patients with low *StAR* expression generally showed high levels of WNT activation signature and of individual WNT target genes expression (*LEF1*, *AXIN2*, *APCDD1*). Altogether, these observations suggested that PKA activity could repress WNT-mediated tumour development in mouse models and in humans.

## Discussion

**Figure 7:**
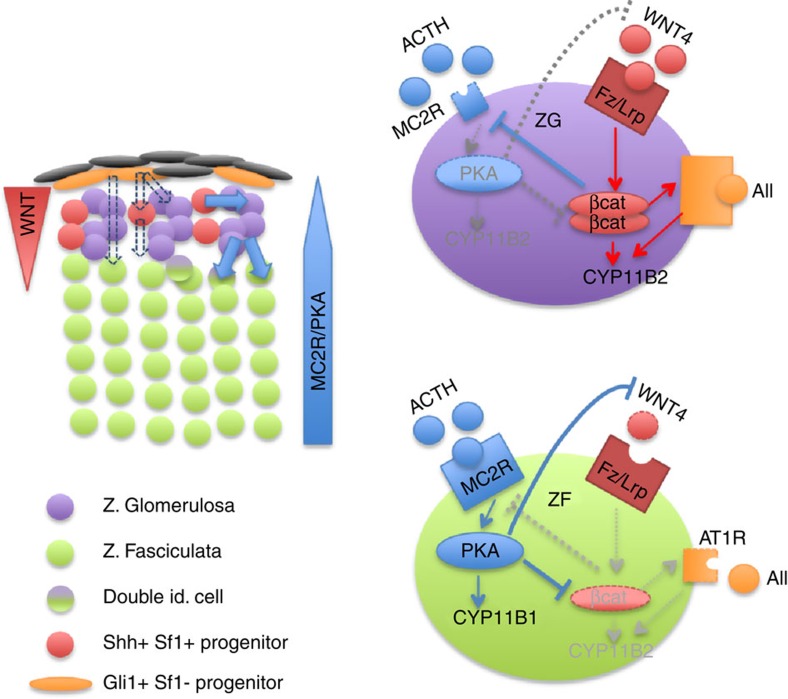
Model for adrenocortical zonal differentiation. Left panel shows the zonal organization of the adrenal cortex with capsular (orange) and subcapsular (red) progenitor cells, ZG cells (purple), ZF cells (green) and transiting cells with double identity (purple and green). Solid blue arrows show the normal process of cell renewal occurring through recruitment of Shh-positive progenitors that initially differentiate as ZG cells and subsequently differentiate as ZF cells as they migrate within the cortex. Dashed arrows show alternative renewal pathways relying on capsular (Shh-negative) progenitors. Right panels show the interplay between WNT and PKA signalling pathways in ZG cells (purple) and ZF cells (green). Red arrows: activation; blue arrows: inhibition; grey arrows: inactive pathway.

Adrenal cortex renewal after recruitment of subcapsular progenitors preferentially occurs through lineage conversion from ZG to ZF cell identity^4^. Here, we show that establishment of ZG identity relies on WNT pathway activation through expression of WNT4, which has recently been proposed to act as a local relay for the effect of capsular RSPO3 on ZG differentiation^34^. We further show that either ACTH treatment or genetic constitutive PKA activation resulting from RIα ablation throughout the adrenal cortex not only inhibits canonical WNT signalling and subsequent glomerulosa differentiation by repressing WNT4 expression, but also allows ZF expansion in the presumptive ZG area. This strongly suggests that PKA activation within inner adrenal cortex is essential to restrict WNT pathway activity, inhibit subsequent ZG differentiation and allow lineage conversion towards ZF differentiation. Altogether, these observations suggest the following integrated model for functional adrenal cortex zonation (Fig. 7): within outer cortex, progenitor cells engage in glomerulosa differentiation in response to active WNT signalling, which stimulates expression of angiotensin II receptor AT1R and of CYP11B2 (ref. 7). The activation of β-catenin also ensures inhibition of ZF differentiation through repression of MC2R and CYP11B1 expression, which maintains cells in their ZG phenotype (this paper and refs 6, 7, 10). Along their centripetal migration, cells with ZG identity progressively escape from active WNT signalling influence, which results in decreased β-catenin activation and extinction of LEF1 expression as they reach ZF (this paper). WNT-mediated inhibition of MC2R expression^10^ is released, which allows for activation of PKA signalling by ACTH. This in turn, definitively inhibits WNT signalling (this paper), which allows for acquisition of fasciculata identity. In this model, rather than an abrupt transition from ZG to ZF identity, cells at the boundary of ZG and ZF would transiently display features of both differentiation programs. The observation of cells with expression of both CYP11B1 and CYP11B2 in the inner glomerulosa is consistent with this hypothesis^4^. This suggests that adrenal cortex zonation and lineage conversion result from a subtle equilibrium between WNT and PKA signalling pathways. This is reminiscent of the role of PKA in neural tube patterning through Hedgehog pathway inhibition^35^, which raises the possibility that such pathways interactions are involved in cell fate decisions and lineage conversion in other tissues.

One apparent contradiction to our model is the lack of ZG expansion in *Mc2r* knockout mice^9^ that would be predicted to result from derepression of β-catenin activity within ZG. However, one likely explanation is that absence of PKA signalling within the cortex of MC2R knockout mice blocks cortical cell renewal, which would prevent centripetal displacement of ZG cells within the cortex. This would then artificially maintain a relatively normal ZG/ZF boundary, even in the absence of Mc2r expression. Consistent with this hypothesis, dexamethasone treatment for 2 weeks was shown to inhibit lineage conversion from ZG to ZF^4^. Another non-mutually exclusive hypothesis is that the restricted domain of WNT4 expression and activity is roughly preserved even in the absence of PKA signalling, which would ensure maintenance of zonation in cases of HPA axis depression. In this scenario, PKA-mediated WNT4 repression would just work as a secondary lock mechanism to ensure normal cortex zonation in a physiological context. This will have to be carefully examined once a conditional *Prkaca* allele becomes available.

In the adrenal, β-catenin activation is associated with both benign and malignant tumorigenesis. In contrast, abnormal PKA signalling has only been described in benign tumours. Observation of abnormal β-catenin accumulation within a number of benign tumours associated with PKA pathway activation suggested that some of the oncogenic effects of PKA could be mediated by WNT pathway activation^36^,^37^,^38^,^39^,^40^. Our data clearly show that this is an unlikely scenario as explained below. (1) There is clear inhibition of WNT pathway activity and β-catenin accumulation in our mouse model of *Sf1:Cre*-mediated *Prkar1a* inactivation. This inhibition can even partially rescue deleterious WNT-induced developmental phenotypes. (2) Combination of PKA activation (through *Prkar1a* inactivation) with WNT pathway activation mediated by the *Akr1b7:Cre* driver, partially inhibits WNT-induced tumorigenesis. (3) Inactivation of one allele of the catalytic subunit of PKA markedly accelerates WNT-induced tumorigenesis. Although we cannot completely rule out that species-specific mechanisms may account for these effects, it is more likely that the kinetics of pathway alterations play an essential role in phenotypic outcome. Indeed, in most PKA-dependent tumours that were analysed, PKA activation resulted from germline mutations of *PRKAR1A* or *GNAS*. Although β-catenin was found activated in these tumours on the basis of immunohistochemical analyses, *CTNNB1* somatic mutations were mostly found in larger tumours or macronodules that developed in the context of micronodular lesions^36^,^38^. Thus in patients, the sequence of events starts with PKA activation, followed by β-catenin activation in the most aggressive tumours. This is in sharp contrast with our mouse models in which both alterations are triggered at the same time by Cre recombinase expression. It is thus tempting to speculate that those tumours that combine both alterations in patients have overcome the negative effect of PKA on β-catenin activity, presumably through mutations in some essential components of this inhibitory mechanism, which would result in a selective growth advantage.

The marked acceleration in WNT-induced tumorigenesis in response to *Prkaca* heterozygosity is intriguing. Indeed, mice combining both alterations develop tumours with malignant characteristics as early as 12 months. This is in striking contrast with the 16 to 19 months period required for β-catenin activation alone, to induce a similar phenotype with the same Cre driver^6^,^41^. It suggests that a decrease in PKA activity could favour acquisition of malignant characteristics in adrenocortical tumours. Consistent with this hypothesis, and even though *StAR* expression may not completely reflect the degree of PKA activity, our data suggest that decreased PKA signalling is associated with more aggressive tumours that exhibit higher levels of WNT target genes expression in ACC patients. This is consistent with published data showing that LOH at the ACTH receptor was associated with more aggressive adrenal cortex carcinoma (ACC) in a small cohort of patients^42^. However, there was no evidence of MC2R LOH in the TCGA cohort, except for one patient (ID: TCGA-OR-A5JY) with homozygous deletion of the locus. This suggests that downregulation of PKA activity may result from alterations of other key players in the pathway or even from increased feedback, resulting from adrenal endocrine overactivity. Although this hypothesis will require thorough analysis of larger cohorts, it suggests that PKA activation suppresses WNT-induced malignant adrenal tumorigenesis. This is in line with the recent finding that PKA can act as a tumour suppressor that prevents basal cell carcinoma formation. However, in the epidermis, PKA exerts its protective effect by restraining Hedgehog and YAP signalling^43^.

Our data showing an inhibitory action of PKA activation on WNT/β-catenin signalling within the adrenal cortex, is in sharp contrast with data from the literature showing that PKA can stimulate WNT pathway activity^17^,^18^,^19^,^20^. Indeed our data show that in contrast with COS7 cells, in which PKA stimulation is associated with induction of *Axin2* expression (ref. 18 and this paper), H295R adrenocortical cells in culture respond to PKA stimulation by accumulation of the inactive phospho-T41/S45 form of β-catenin even though activating phosphorylation at serine 552 is induced as efficiently as in COS7 cells. This phenomenon is also observed *in vivo*. Indeed *Sf1:Cre;Prkar1a Fl/Fl* adrenals show a combination of increased inactivating (pT41/S45) and decreased activating β-catenin phosphorylation (pS552), which results in decreased accumulation of the active ABC form of the protein. Whether increased T41/S45 is a direct effect of PKA on β-catenin in adrenocortical cells is unclear, even though experimental evidence suggests that these residues could be targets of the catalytic activity of PKA, both in reconstructed *in vitro* kinase assays and in cell culture^19^. Another possibility, at least *in vivo*, is that PKA indirectly triggers activating Y216 phosphorylation of GSK3β (Fig. 2d–w), which would result in increased T41/S45 phosphorylation of β-catenin. Beyond regulation of critical phosphorylations, our unbiased microarray analysis of WNT pathway regulators expression in *Sf1:Cre;Prkar1aFl/Fl* adrenals suggests that PKA may exert its inhibitory effect at multiple levels of WNT signalling pathway by deregulating expression of a number of potential repressors and activators. In this paper, we provide genetic evidence that inhibition of WNT4 expression in response to PKA signalling activation is one important aspect of this phenomenon. Indeed, *Sf1:Cre;Wnt4Fl/Fl* mutants display a differentiation phenotype reminiscent of *Sf1:Cre;Prkar1aFl/Fl* mutants and Lef1 repression by ACTH is almost abrogated in *Wnt4* mutants. One conundrum of our study is the observation that PKA activation is still capable of inhibiting WNT signalling in ΔCat mice (Figs 4 and 5 and Supplementary Fig. 8) and H295R cells (Fig. 2) that express a destruction-complex resistant β-catenin^44^. In this context, WNT signalling is considered to be largely independent of WNT ligands. However, experimental evidence suggests that cells with activated β-catenin retain some sensitivity to extra-cellular signals such as RSPO2 and SFRPs^45^,^46^. Consistent, with a potential role of WNT4 inhibition in this setting, we observed a marked reduction in its accumulation in ΔCat mice, in response to ACTH treatment (Supplementary Fig. 8). Another non-mutually exclusive hypothesis could involve the potential repressor CDC73/parafibromin, a component of RNA-Pol II-associated PAF1 complex. It had initially been described as a positive regulator β-catenin transcriptional activity by recruiting Pygopus to β-catenin^25^. However, this positive interaction is dependent on CDC73 tyrosine dephosphorylation by the phosphatase SHP2 (ref. 47) and recent evidence suggests that CDC73 can repress WNT signalling in colorectal cancer cells and B-cells in response to activation of BTK (Bruton's Tyrosine kinase)^26^, a kinase that has been proposed to activate PKA^48^. Our data show that CDC73 is normally accumulated in the nucleus of ZF but not ZG cells (Fig. 3c). Interestingly, we further show that this expression pattern expands within presumptive ZG upon deletion of *Prkar1a* (Fig. 3c). This strongly suggests that CDC73 may directly interact with and repress β-catenin transcriptional activity in response to PKA stimulation within the adrenal cortex. Careful evaluation of this hypothesis may reveal novel relevant interactions that could be targeted to block WNT pathway activation in adrenocortical tumours.

In conclusion, we have identified PKA-mediated WNT inactivation as an essential mechanism for lineage conversion from ZG to ZF and maintenance of adrenal cortex zonation. Our genetic data further show that this novel mechanism is also a potentially important repressor of WNT-induced tumorigenesis in the adrenal cortex.

## Methods

### Mice

All animal studies were approved by Auvergne Ethics committee and were conducted in agreement with international standards for animal welfare. *Akr1b7:Cre* (stochastic Cre expression in steroidogenic cells of the adrenal cortex starting t E14.5)^49^, *Sf1:Cre* (Cre expression in all steroidogenic cells of the adrenal cortex from its inception)^14^, *mT/mG* (recombination reporter system introduced at the ROSA26 locus that allows a switch from constitutive Tomato fluorescence to GFP upon recombination)^15^, *Ctnnb1Fl(ex3)* (Floxed allele of *Ctnnb1* that allows Cre-mediated deletion of the third exon of the gene and subsequent constitutive activation)^30^, *Prkar1aFl/Fl* (Floxed allele of *Prkar1a* that allows Cre-mediated inactivation)^13^, *Prkaca+/−* (constitutive heterozygous deletion of *Prkaca*)^50^*, Wnt4Fl/Fl* (Floxed allele of *Wnt4* that allows Cre-mediated inactivation)^29^, TopGal mice (Canonical WNT pathway activation reporter mice)^12^ were all previously described. They were all maintained and bred on a mixed background. Throughout the manuscript, ΔCat refers to either heterozygous *Ctnnb1Fl(ex3)/+* or homozygous *Ctnnb1Fl(ex3)/Fl(ex3)* mice. We did not observe any discernible difference between both genotypes, which is consistent with the dominant effect of the mutation^30^. Littermate control animals were used in all the experiments. All analysed mice were female. *Prkar1aFl/Fl*;*Sf1:Cre and ΔCat;Prkar1aFl/Fl*;*Sf1:Cre* mice were analysed from 4 to 6 weeks. *ΔCat;Prkar1aFl/Fl*;*Akr1b7:Cre* and *Wnt4Fl/Fl;Sf1:Cre* mice were analysed at 6 months. *ΔCat;Prkaca+/−;Akr1b7:Cre* mice were analysed at 12 months. For hormonal manipulations, 6-month-old C57Bl/6 mice were injected subcutaneously with vehicle for 5 days (sesame oil twice daily), dexamethasone actetate for 5 days, which depletes endogenous ACTH production (75 μg twice daily in sesame oil) or dexamethasone actetate for 3 days and Synacthene Retard for 2 days (long-lasting exogenous source of ACTH, I.M, 1.2 U, Sigma Tau Laboratories). *Sf1:cre Wnt4Fl/Fl* mice and littermate controls were treated for 3 days with Synacthene Retard (1.2 U). For acute ACTH stimulation, wild-type mice were treated for 24 h with Synacthene Retard (1.2 U). At the end of the experimental procedures, the mice were killed by decapitation and blood was collected in vacuum blood collection tubes (VF-053STK, Terumo). The adrenals were either frozen in liquid nitrogen or fixed in 4% PFA. Total mRNAs were extracted using RNAII nucleotide extraction kit (Macherey Nagel) according to the manufacturer's instructions. Total proteins were extracted with RIPA buffer in the presence of protease (Complete 1X, Roche) and phosphatase inhibitors (NaF, 1 mM; Na_3_VO_4_, 1 mM).

### Immunohistochemistry

Immunohistochemistry for β-catenin (1/500, BD 610153), DAB2 (1/500, BD 610464), CYP11B2 (1/200, gift from C. Gomez-Sanchez, University of Mississippi, USA), AKR1B7 (1/500, ref. 6), Ki67 (1/200, RM9106-SO, Thermo Fisher) and 20αHSD (1/20000, kind gift from Y. Weinstein, Ben-Gurion University, Israel) was performed on tissues embedded in paraffin, after unmasking with sodium citrate 10 mM, Tween 0.05% (β-catenin, AKR1B7, Ki67 and 20αHSD) or Tris 10 mM, EDTA 1 mM, pH 9.0, followed by 5 min in 10%SDS for CYP11B2, as previously described^6^,^16^,^41^. For LEF1 immunodetection, the slides were treated for 20 min in boiling sodium citrate 10 mM, tween 0.05% and blocked with 1% BSA. They were then incubated overnight with LEF1 antibody (1/100, #04-1159, Millipore) in 0.1% BSA. For immunodetection of NDRG1 (1/500, Ab37897, Abcam), CDC73 (1/500, SC-33638, Santa Cruz Biotechnology) and WNT4 (1/200, Ab91226, Abcam), the slides were treated for 20 min in boiling Tris 10 mM, EDTA 1 mM, pH 9.0 and blocked with 1% BSA. They were then incubated overnight with NDRG1, CDC73 and WNT4 antibodies. All of the above primary antibodies were detected with SignalStain Boost HRP-Polymer solution (#8114S or #8125P, Cell Signalling) and either Vectastain ABC (PK-4000, Vector Labs) or TSA-Alexa 488 (T20948, Thermo Fisher) as substrates. For GFP immunodetection, the slides were treated for 20 min in boiling Vector Unmasking Solution (H3300, Vector Labs) and blocked with 10% foetal bovine serum (FBS) and 3% bovine serum albumin (BSA). They were then incubated overnight with GFP antibody (1/200, Ab5450, Abcam) in FBS 1%, BSA 1%. Primary antibody was detected with anti-goat Alexa 488 antibody (1/1,000, A11055, Molecular Probes Thermo Fisher). All immunohistochemical analyses were conducted on an automated processor (Intavis InSitu Pro) to ensure homogeneity and reproducibility of detections. Images were acquired with a Zeiss Axioplan 2 microscope and Axiocam HR camera. They were minimally processed for global levels and white balance using Adobe Photoshop. Image settings and processing were identical across genotypes.

### X-Gal staining

X-Gal staining was performed on whole adrenals as described in ref. 49, after fixation in 0.2% glutaraldehyde for 1 h. After rinsing in wash buffer (phosphate-buffered saline (PBS), 2 mM MgCl_2_, 0.1% Na deoxycholate, 0.02% NP40, 0.05% BSA), tissues were incubated for 12 h in X-Gal staining solution (PBS, 0.02% Nonidet-P40, 2 mM MgCl_2_, 5 mM K_3_Fe(CN)_6_, 5 mM K_4_Fe(CN)_6_, 1 mg ml^−1^ X-gal) at 37 °C. After staining, the adrenals were rinsed in PBS and fixed in 4% PFA for 1 h. The tissues were then cryoprotected in sucrose 20% in PBS overnight at 4 °C and embedded in OCT. Ten micrometre sections were cut on a cryostat and deposited on Superfrost slides for observation.

### RT–qPCR analysis

One microgram of total mRNAs (from tissues or cell culture) was reverse transcribed for 1 h at 37 °C with 5 pmol of random hexamers primers, 200 units reverse transcriptase (M-MLV RT, M1701, Promega), 2 mM dNTPs and 20 units RNAsin (N2615, Promega). One microlitre of a one-tenth dilution of cDNA was used in each qPCR. Except for *VegfA* and *Connexinα43* that were amplified with Taqman chemistry as described in ref. 41, all other reactions were conducted with SYBR qPCR Premix Ex Taq II Tli RNase H+ (TAKRR820W, Takara). Primer pairs are listed in Supplementary Data 3. For each experiment and primer pairs, efficiency of PCR reactions was evaluated by amplification of serial dilutions of a mix of cDNAs. Relative gene expression was obtained by the Δ*ΔCt* method after normalization to *36b4* (mouse) or *PPIB* (human).

### Intra-adrenal aldosterone evaluation

Whole adrenal glands were disrupted in 1X PBS with tungsten beads (Qiagen) using a Tissue Lyser (Qiagen). Adrenal extract was then centrifuged at 12,000*g* and steroids contained in the supernatant were extracted with 10 volumes of dichloromethane. After evaporation, the samples were resuspended in 150 μl of buffer and aldosterone concentration was measured with Aldosterone ELISA Kit (CAN-ALD-450, Diagnostics Biochem Canada).

### Cell culture

Human adrenocortical cancer H295R cell line (obtained from ATCC as a mycoplasma free cell line) was grown with DMEM/Ham's F12 supplemented with 10% FBS (S1800-500, Biowest), 2 mM L-Glutamine (25030, Gibco), 50 U ml^−1^ penicillin, 100 μg ml^−1^ streptomycin and 1X insulin transferrin selenium (41400-045, Gibco). COS7 cell line was grown with DMEM supplemented supplemented with 5% FBS (S1800-500, Biowest), 2 mM L-Glutamine (25030, Gibco), 50 U ml^−1^ penicillin and 100 μg ml^−1^ streptomycin. For PKA stimulation treatments, the cells were seeded at a density of 3 × 10^5^ cells per well in six-well plates. The day after seeding, the cells were deprived of serum and growth factors for 12 h. They were then treated with vehicle (dimethyl sulfate (DMSO)) or with Forskolin (10^−5^ M) for the indicated amount of time. For inhibition of PKA signalling, H295R cells were deprived of serum and growth factors for 12 h. They were then treated with vehicle (DMSO), 10 μM H89, 10^−5^ M Forskolin or a combination of 10 μM H89 and 10^−5^ M Forskolin for 8 h, following 2 h pre-incubation with H89. For transfection experiments, the cells were seeded at a density of 3 × 10^5^ cells per well and transfected with 1 μg pCMV-WNT4 or empty vector (pCMV5) with Effectene reagent (Qiagen) according to the manufacturer's instructions. Twenty-four hours after transfection, the cells were deprived of serum and growth factors for 12 h and treated as above. Proteins were extracted with RIPA buffer in the presence of protease (Complete 1X, Roche) and phosphatase inhibitors (NaF, 1 mM; Na3VO4, 1 mM). mRNAs were extracted by TriReagent (Molecular Research Center Inc.) according to the manufacturer's instructions. All the experiments were performed at least three times in triplicate.

### Western blot

Ten to forty micrograms of total proteins were loaded on 8% SDS–PAGE gel, transferred onto nitrocellulose and detected with the following antibodies: phospho-β-catenin S552 (1/1,000, #5651, CST), phospho-β-catenin S675 (1/1,000, #4176, CST), phospho-β-catenin T41/S45 (1/1,000, #9565, CST), phospho-β-catenin S33/37/T41 (1/200, #9561, CST), active β-catenin ABC (1/1,000, 05-665, Millipore), β-catenin (1/1,000, 610153, BD), phospho-GSK3 (1/1,000, 9331s, CST), GSK3β (1/1,000, 9315s, CST), phospho-GSK3β Y216 (1/1,000, Ab75475, Abcam) and GAPDH (1/1,000, NB30021, Novus). The signals were quantified with a DNR MF ChemiBis 3.2 camera system and Multi Gauge software suite (Fujifilm). Expression of the phospho-proteins was normalized to expression of the corresponding total protein. Full western-blot pictures are provided as Supplementary Fig. 9.

### Microarray analysis of gene expression

Adrenal gene expression profiles for four 6-week-old *Sf1:Cre;Prkar1aFl/Fl* and four wild-type littermates were analysed using Affymetrix Mouse Gene 2.0 ST Arrays (Raw and processed data are deposited on NCBI GEO platform). Gene expression was normalized by RMA (Affy R package) and genotype comparisons were performed with *t*-test. All the *P* values were adjusted by the Benjamini–Hochberg correction method. Volcano plot was generated with R and displays −Log10(adjusted *P* value) as a function of Log2(fold change in knockout versus WT). Genes with adjusted *P* value <0.05 and Log 2 fold changes <−1 (blue dots) or >1 (red dots) were considered significantly down and upregulated, respectively. The WNT pathway targets and regulators list was curated from WNT Homepage (http://web.stanford.edu/group/nusselab/cgi-bin/wnt/) and Pubmed searches. They are appended to this manuscript as Supplementary Data 1. Heatmaps were generated with R and represent colour-coded individual median centred gene expression levels (in the Log2 space) in four WT and four knockout adrenals. Genes were ordered according to the mean fold change (Log2) in knockout versus WT. Only genes with adjusted *P* values <0.05 are shown in these representations.

### Patients' data

Gene expression and clinical data from adrenocortical carcinoma patients were extracted from mRNA sequencing data available from Broad Institute GDAC Firehose (TCGA data version 2015_03_26, http://gdac.broadinstitute.org). These include 79 adrenal cortex carcinomas. All the calculations were performed on Log2 values of RSEM normalized read counts. Mutational status was retrieved from cBioPortal (http://cbioportal.org). Heatmaps were generated with R. WNT gene signature was determined as the geometric mean of the Log2 values of RSEM normalized read counts for WNT target genes *LEF1*, *AXIN2* and *APCDD1*.

### Statistical analyses

Statistical analyses of biological data were performed with R and GraphPad Prism 5.

### Data availability

The authors declare that all data supporting the findings of this study are available within the article and its Supplementary Information files or from the corresponding author upon reasonable request. Microarray data are deposited on Gene Expression Omnibus repository with reference GSE77630.

## Additional information

**How to cite this article:** Drelon, C. *et al*. PKA inhibits WNT signalling in adrenal cortex zonation and prevents malignant tumour development. *Nat. Commun.* 7:12751 doi: 10.1038/ncomms12751 (2016).

## Supplementary Material

Supplementary Figures Supplementary Figures 1-9

Supplementary Data 1 WNT signalling pathway targets and regulators. A list of 127 canonical WNT signalling targets and regulators was curated from the WNT homepage (http://web.stanford.edu/group/nusselab/cgi-bin/wnt/) and Pubmed searches.

Supplementary Data 2 Genes significantly down and up-regulated in Sf1:Cre; Prkar1a Fl/Fl adrenals.

Supplementary Data 3 Sequences of the primers used for RTqPCR
