# Supplementary material for: PKA inhibits WNT signalling in adrenal cortex zonation and prevents malignant tumour development
Source: Nat Commun. 2016 Sep 14;7:12751. doi: 10.1038/ncomms12751 (PMC5027289; doi:10.1038/ncomms12751)
Supplement: Supplementary Figures — 1-9 [file ncomms12751-s1.pdf]

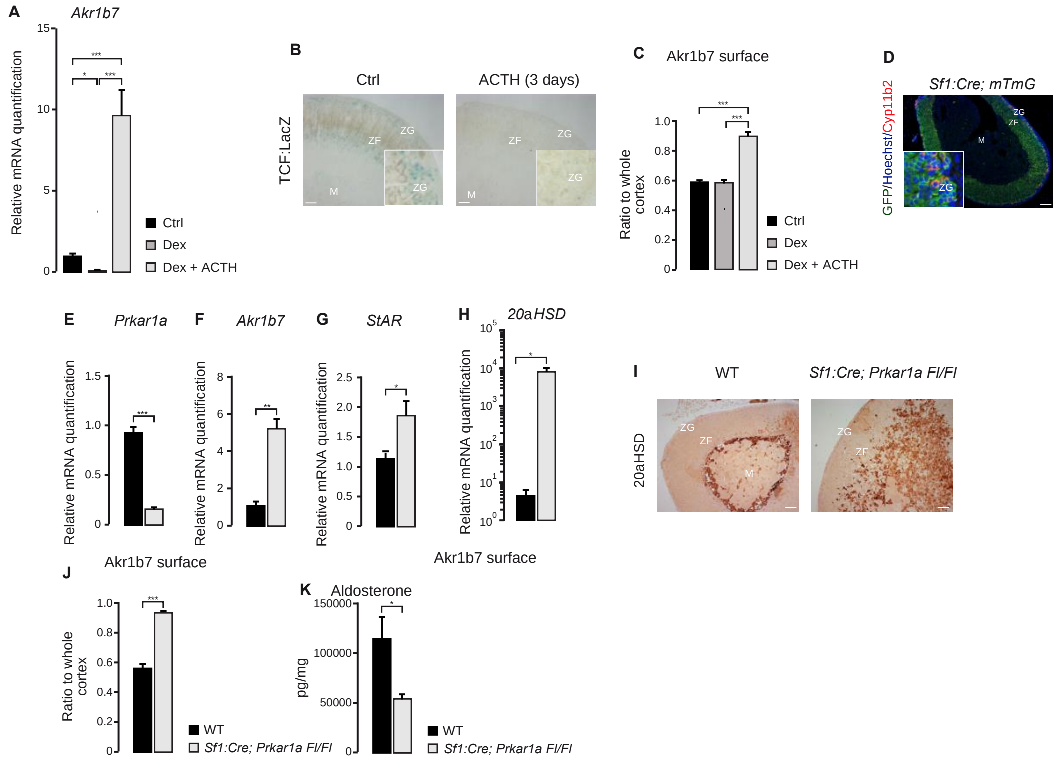

Supplementary Figure 1. Effect of PKA stimulation on functional adrenal differentiation. A- Dexamethasone and ACTH treatments modulate *Akr1b7* expression. Wild-type C57Bl/6 mice were treated for 5 days with vehicle (Ctrl) or Dexamethasone (Dex) or for 3 days with dexamethasone followed by two days of Synacthene retard (Dex + ACTH). *Akr1b7* expression levels were then analysed by RTqPCR on mRNAs extracted from adrenals. Bars represent mean expression in 4 animals per group ± SEM. B- ACTH treatment inhibits activity of the WNT pathway reporter TopGal. Transgenic mice bearing the WNT reporter gene TopGal were treated for three days with vehicle (Ctrl) or ACTH (synacthene retard) and reporter activity was detected by X-Gal staining. Inset shows a high magnification picture of ZG. C- ACTH treatment increases AKR1B7 staining area in the adrenal cortex of wild-type mice. Wild-type C57Bl/6 mice were treated for 5 days with vehicle (Ctrl) or Dexamethasone (Dex) or for 3 days with dexamethasone followed by two days of Synacthene retard (Dex + ACTH). AKR1B7 expression was detected by immunohistochemistry and stained areas were measured and expressed as a percentage of total adrenal cortex surface. Bars represent the mean of 4 adrenals per treatment group ± SEM. D- *Sf1:Cre* induces LoxP recombination in all cortical cells. *Sf1:Cre* mice were mated with *mT/mG* reporter mice to generate *Sf1:Cre;mTmG* offspring. Recombination efficiency was analysed by immunodetection of GFP expression. Sections were also stained with Cyp11b2 (ZG) and Hoechst (nuclei). Recombination was induced in all cortical cells including ZG (inset). E- *Prkar1a* expression is decreased in *Sf1:Cre; Prkar1a Fl/Fl* mice. *Prkar1a* expression levels were analysed by RTqPCR on mRNAs extracted from wild-type and *Sf1:Cre;Prkar1aFl/Fl* adrenals. F-G-H *Prkar1a* ablation induces expression of *Akr1b7*, *StAR* and *20αHSD*. *Akr1b7, StAR and 20αHSD* expression levels were analysed as above. I- 20αHSD expression domain is expanded in *Sf1:Cre Prkar1aFl/Fl* mice. 20αHSD expression was analysed by immunohistochemistry in 2.5 month-old nulliparous wild-type and *Sf1:Cre;Prkar1aFl/Fl* female mice. J- Akr1b7 staining area is increased in the adrenal cortex of *Sf1:Cre;Prkar1a Fl/Fl* mice. AKR1B7 staining in wild-type and *Sf1:Cre;Prkar1aFl/Fl* mice was evaluated as in C. K- Intra-adrenal aldosterone concentration is reduced in *Sf1:Cre;Prkar1aFl/Fl* mice. Intra-adrenal aldosterone concentration was evaluated by ELISA after steroids extraction from whole adrenal gland tissue. In E-H and I, bars represent mean expression in 6 animals per group ± SEM. Statistical analyses were conducted by one-way ANOVA followed by Tukey's post-hoc test (A,C) or Student's *t* test (E-H, J-K). * $p<0.05$; ** $p<0.005$; *** $p<0.0005$. Scale bars represent 100 μm. ZG, zona glomerulosa; ZF, zona fasciculata; M, medulla.

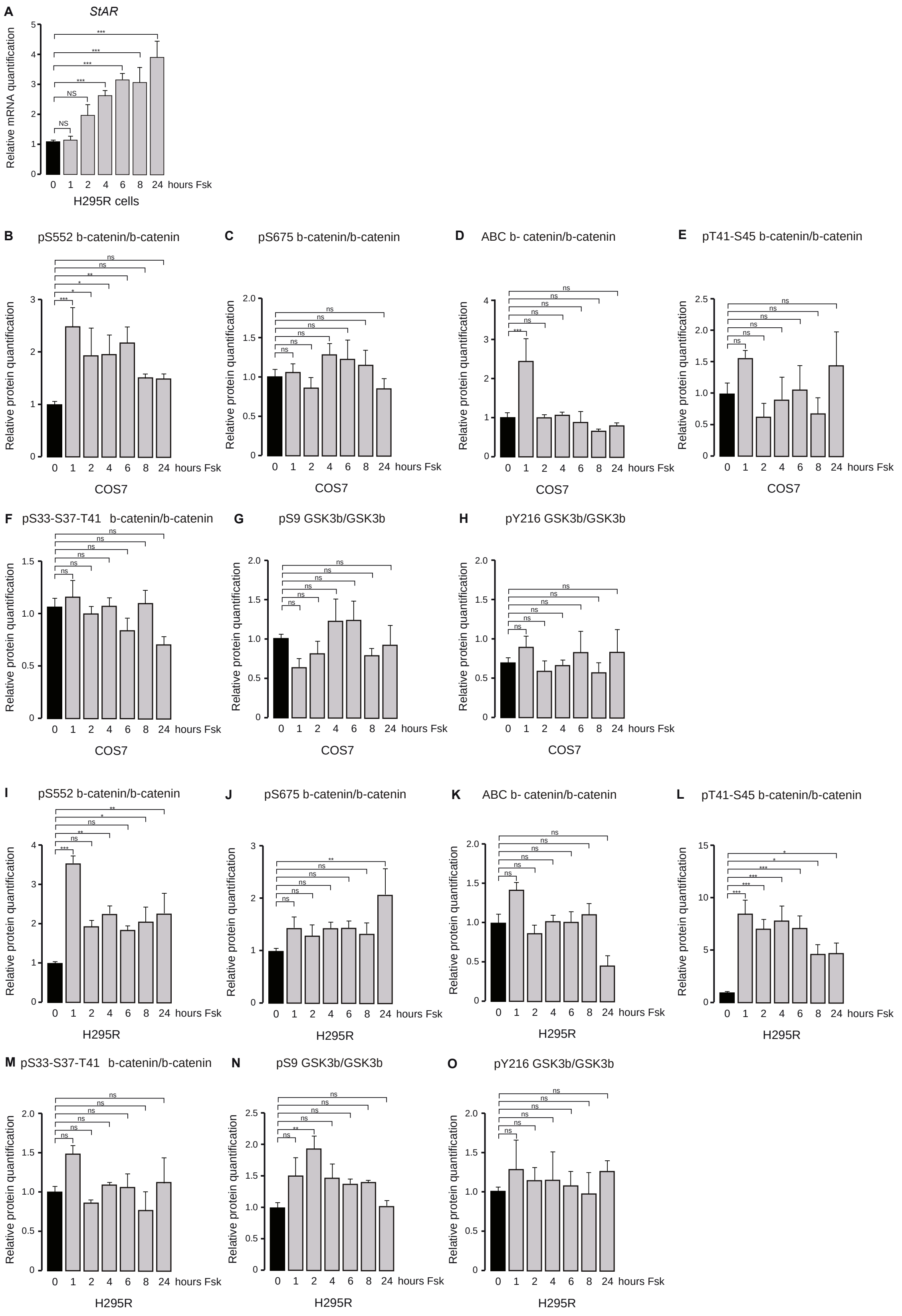

Supplementary Figure 2. Effect of PKA stimulation on WNT pathway phosphorylations. A- *StAR* expression is induced by forskolin in H295R cells. Human H295R cells were treated with either vehicle (0) or Forskolin 10^{-5}M (Fsk) for 1 to 24 h and *StAR* expression was analysed by RTqPCR. Bars represent mean expression in at least three independent experiments (in triplicate) ± SEM. **B-O- Quantification of WNT pathway regulators phosphorylation in COS7 cells (B-H) and H295R cells (I-O).** Western-blot signals presented in Fig. 2C were quantified and accumulation of phospho-proteins was normalised with accumulation of corresponding total proteins. Bars represent the mean of fold activation over DMSO (at least 4 independent experiments) for each time point ± SEM. In all experiments, statistical analyses were conducted by one-way ANOVA followed by Dunett's post-hoc test. * p<0.05; ** p<0.005; *** p<0.0005; ns: Not significant.

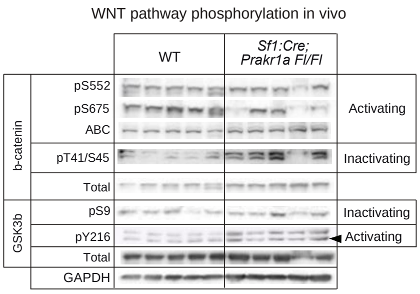

Supplementary Figure 3. Phosphorylation of β-catenin and GSK3β in response to constitutive PKA activation *in vivo*. Activating and inactivating phosphorylations of β-catenin and GSK3β were analysed in adrenal protein extracts from 5 wild-type and 5 *Sf1:Cre; Prkar1a Fl/Fl* mice, independent from samples shown in main manuscript Fig. 2D.

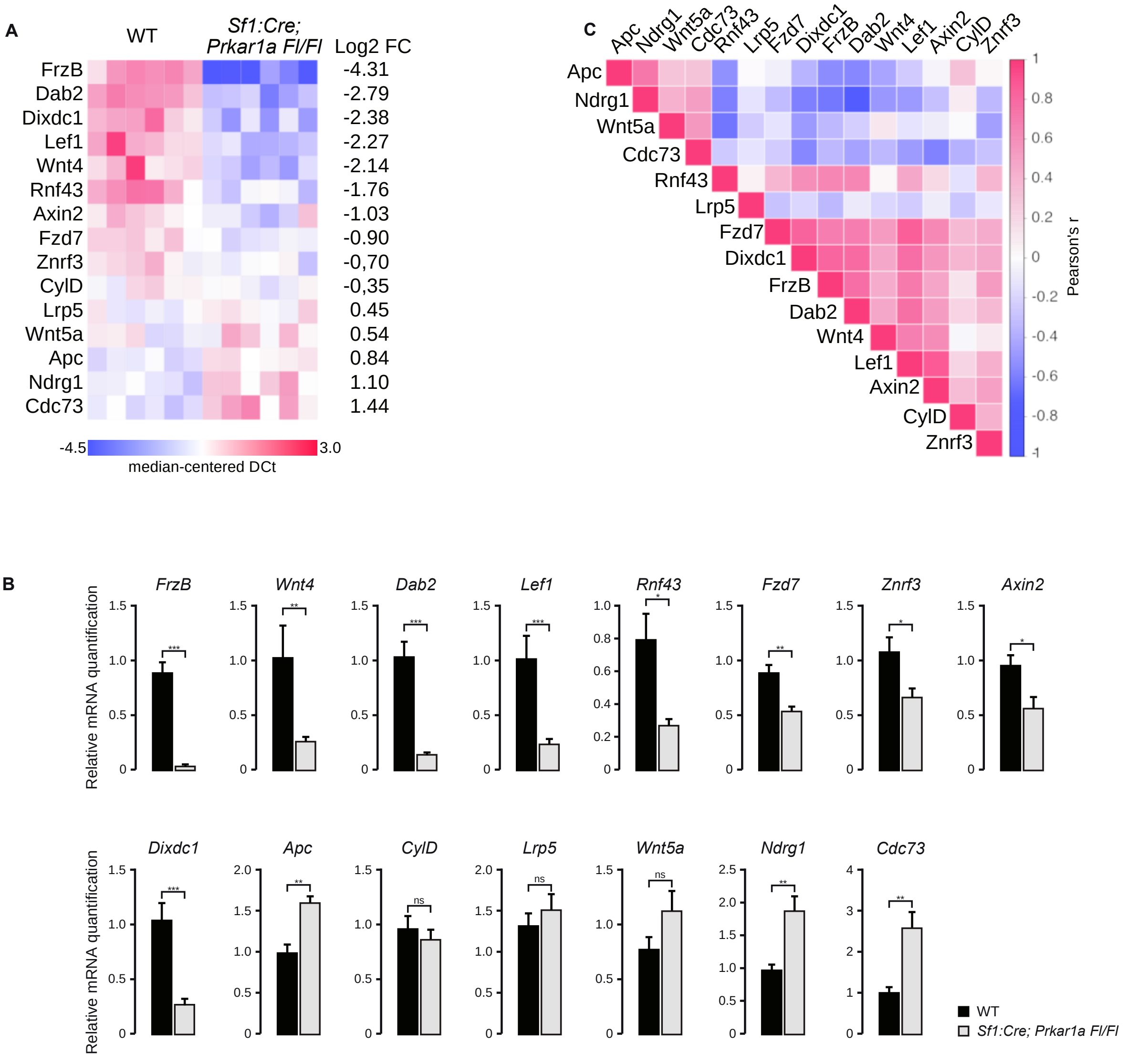

Supplementary Figure 4. Analysis of selected WNT pathway regulators expression in *Sf1:Cre; Prkar1a Fl/Fl* mice by RTqPCR. A- Heatmap representation of RTqPCR data (Log2) in 6 wild-type and 6 *Sf1:Cre; Prkara1a Fl/Fl* mice (independent from the animals used in Fig 3A). B- Representation of RTqPCR data as barplots, following conversion of Log2 values to linear values (2^-ΔΔCt). C- Correlogram generated from RTqPCR data, showing Pearson's correlation coefficients between the 15 WNT pathway genes. Statistical analyses in B were performed by Student's t test. * p<0.05; ** p<0.005; *** p<0.0005; ns: Not significant.

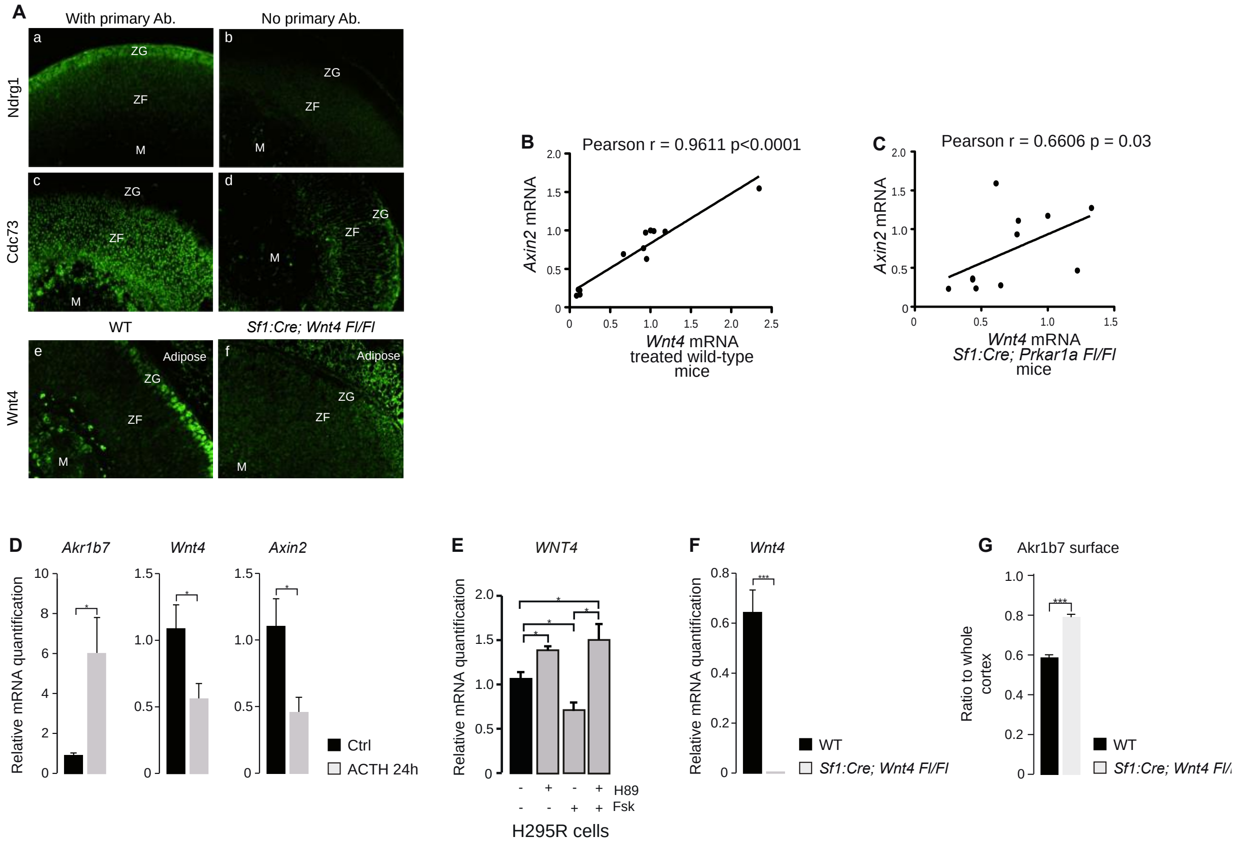

Supplementary Figure 5. Expression of WNT pathway regulators in response to PKA stimulation. A- Evaluation of the specificity of antibodies detecting NDRG1, CDC73 and WNT4. Top two panels show immunodetection of NDRG1 and CDC73 in wild-type adrenals (a,c) and the corresponding negative controls (b,d, no primary antibody). Although some non-specific vascular staining is present in d, specific nuclear staining for CDC73 is undetectable in the absence of the primary. Lower panel shows immunodetection of WNT4 in a wild-type adrenal (e) and the absence of signal in *Sf1:Cre;Wnt4 Fl/Fl* adrenal cortex (f). B-C- *Axin2* and *Wnt4* expression levels are correlated *in vivo*. *Axin2* and *Wnt4* expression levels were correlated on the basis of RTqPCR data presented in Fig 3 D & E. D- Short-term ACTH treatment induces *Akr1b7* and represses *Wnt4* and *Axin2* expression in vivo. Wild-type mice were treated with a single injection of ACTH (Synacthene Retard) or vehicle and sacrificed 24h later. Expression of *Akr1b7*, *Axin2* and *Wnt4* was then evaluated by RTqPCR. Bars represent the mean of expression in 4 animals per treatment group ± SEM. E- H89 treatment induces *WNT4* expression and blocks the inhibitory effect of Forskolin. H295R cells were treated with vehicle (DMSO), forskolin (10^{-5}M), H89 (10 µM) or a combination of both for 8h (after 2h preincubation with H89) and expression of *WNT4* was analysed by RTqPCR. Bars represent the mean expression in three independent experiments ± SEM. F- *Wnt4* expression is almost abolished in *Sf1:Cre;Wnt 4Fl/Fl* adrenals. *Wnt4* expression was analysed by RTqPCR on mRNAs extracted from wild-type and *Sf1:Cre*;*Wnt4Fl/Fl* adrenals. Bars represent the mean expression in 10 adrenals per genotype ± SEM. F- Akr1b7 staining area is increased in the adrenal cortex of *Sf1:Cre;Wnt4 Fl/Fl* mice. AKR1B7 staining in wild-type and *Sf1:Cre;Wnt4 Fl/Fl* mice was evaluated as in Supplementary Fig. 1C. Statistical analyses were performed by one-way ANOVA followed by Dunnett's multiple comparison test (E) or with Student's *t* test with Welch correction (D, F-G). * p<0.05; ** p<0.005; *** p<0.0005; NS: Not significant. ZG, zona glomerulosa; ZF, zona fasciculata; M, medulla.

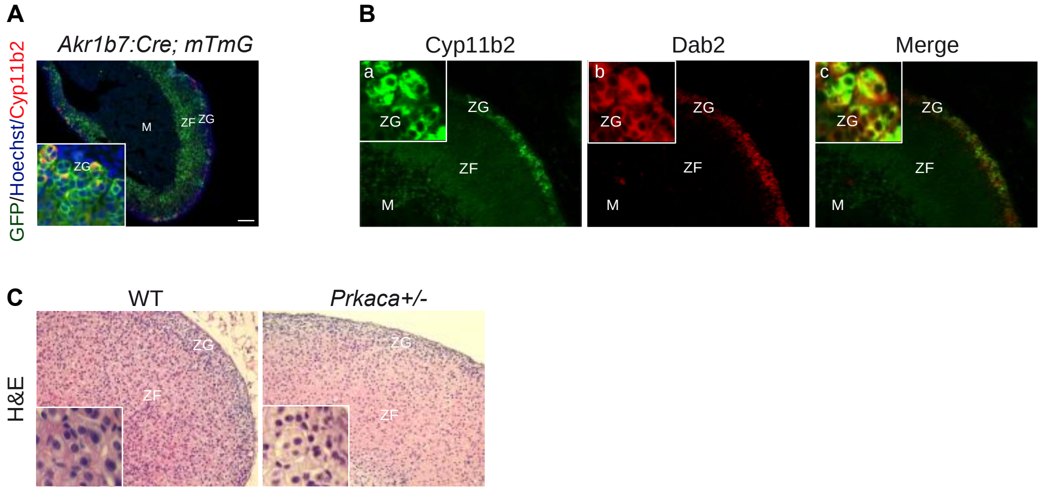

Supplementary Figure 6. *Akr1b7:Cre*-mediated recombination, DAB2 expression in the adrenal and analysis of *Prkaca+/-* adrenals. A- *Akr1b7:Cre* induces LoxP recombination in a subset of cortical cells. *Akr1b7:Cre* mice were mated with *mT/mG* reporter mice to generate *Sf1:Cre;mTmG* offspring. Recombination efficiency was analysed by immunodetection of GFP expression. Sections were also stained with CYP11B2 (ZG) and Hoechst (nuclei). Recombination was induced in a large number of but not all cortical cells, including areas of ZG (inset). B- DAB2 is a *bona fide* ZG marker. Wild-type adrenals were stained with both CYP11B2 (a) and DAB2 (b) antibodies. Panel c shows the overlap between the two signals in ZG (insets). **C- Heterozygosity for *Prkaca* has no obvious impact on adrenal histology.** Adrenals from *Prkaca+/+* (WT) and *Prkaca+/-* were stained with heamtoxylin and eosin. Inset shows high magnification of ZG. ZG, zona glomerulosa; ZF, zona fasciculata; M, medulla.

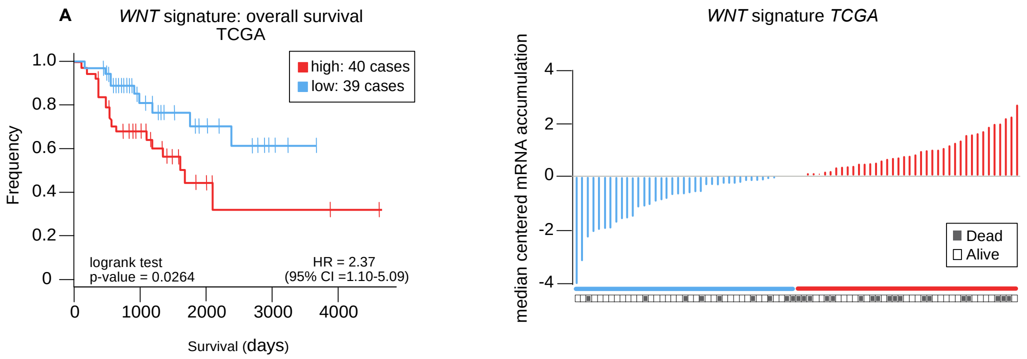

Supplementary Figure 7. Activation of WNT pathway is associated with decreased overall survival in ACC patients. *WNT* activation signature was calculated as the geometric mean of *AXIN2*, *LEF1* and *APCDD1* expression levels that were extracted from mRNA sequencing data from TCGA. Patients were separated as two equivalent groups of high (n=39) and low (n=40) *WNT* activation signature. Left panel shows the Kaplan-Meier estimates of overall survival. In the right panel, patients were ordered by cut-off-centred *WNT* activation signature. Dark grey boxes indicate specific death.

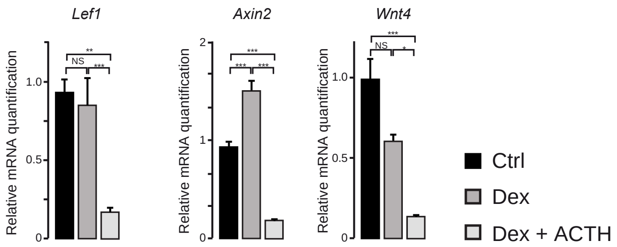

Supplementary Figure 8. ACTH treatment inhibits WNT signalling in ΔCat mouse adrenals. ΔCat mice that express a constitutive allele of *Ctnnb1* in the adrenal cortex [6] were treated for 5 days with vehicle (Ctrl) or Dexamethasone (Dex) or for 3 days with dexamethasone followed by two days of Synacthene retard (Dex + ACTH). *Lef1, Axin2* and *Wnt4* expression levels were then analysed by RTqPCR on mRNAs extracted from adrenals. Bars represent mean expression in 4 animals per group ± SEM. Statistical analysis was performed with Student's *t* test. * p<0.05; ** p<0.005; *** p<0.0005.

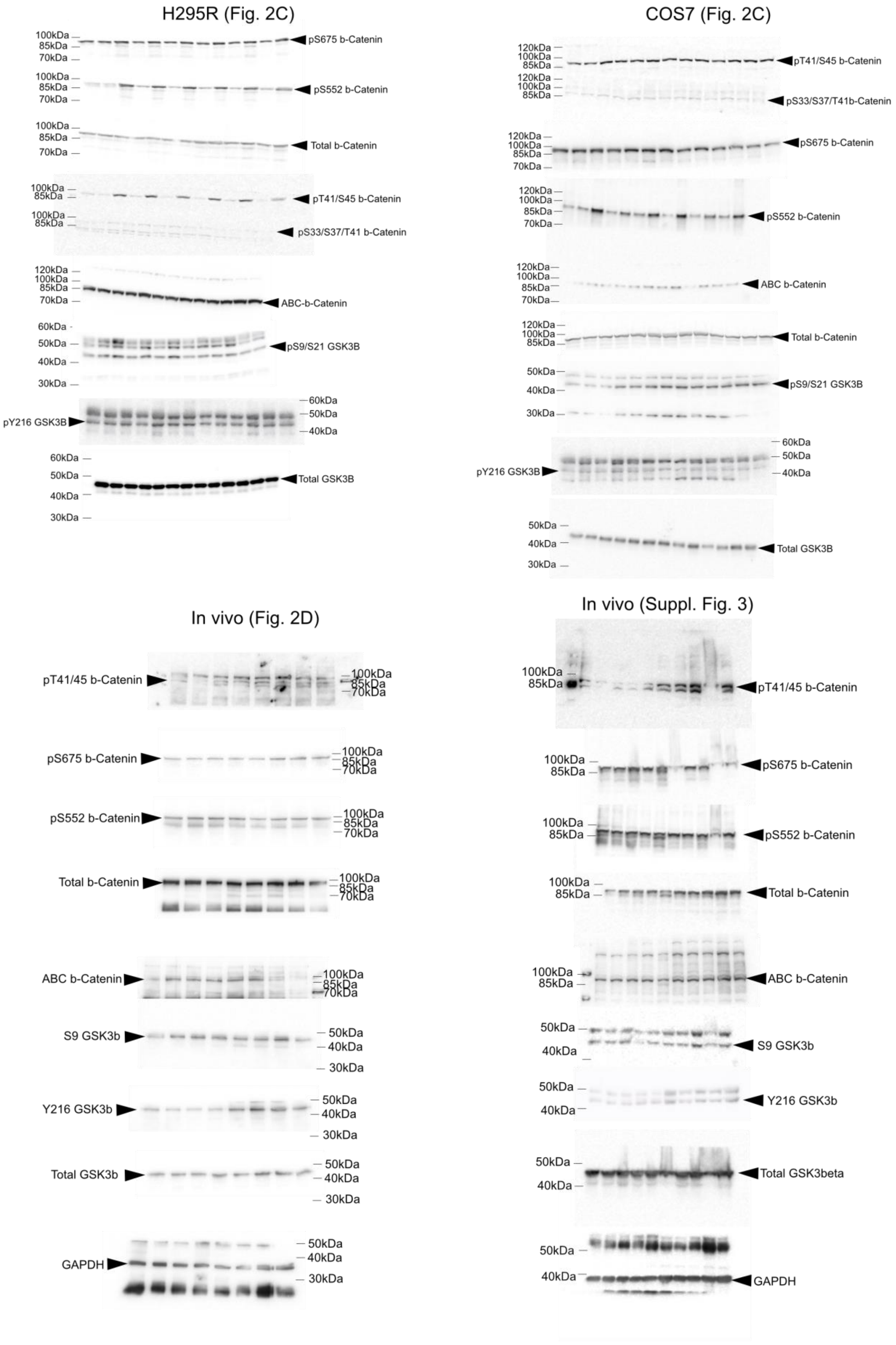

Supplementary Figure 9. Non-cropped versions of the western blots shown in Figure 2C, 2D and Supplementary Figure 3.
